# Activation of the JNKs/ATM-p53 axis is indispensable for the cytoprotection of dermal fibroblasts exposed to UVB radiation

**DOI:** 10.1038/s41419-022-05106-y

**Published:** 2022-07-25

**Authors:** Eleni Mavrogonatou, Maria Angelopoulou, Sophia V. Rizou, Harris Pratsinis, Vassilis G. Gorgoulis, Dimitris Kletsas

**Affiliations:** 1 Laboratory of Cell Proliferation and Ageing, Institute of Biosciences and Applications, National Centre for Scientific Research “Demokritos”, 15341 Athens, Greece; 2 Molecular Carcinogenesis Group, Department of Histology and Embryology, Medical School, National and Kapodistrian University of Athens, 11527 Athens, Greece; 3 Biomedical Research Foundation, Academy of Athens, Athens, Greece; 4 Faculty of Biology, Medicine and Health Manchester Cancer Research Centre, Manchester Academic Health Sciences Centre, University of Manchester, Manchester, UK; 5 Center for New Biotechnologies and Precision Medicine, Medical School, National and Kapodistrian University of Athens, Athens, Greece; 6 Ninewells Hospital and Medical School, University of Dundee, Dundee, UK

**Keywords:** Stress signalling, Cell death

## Abstract

Although UVB radiation is mainly absorbed by the epidermis, ~5–10% of its photons reach and affect the upper part of the dermis. Physiologically relevant UVB doses, able to provoke erythema, induce apoptosis in human dermal fibroblasts in vitro, as well as in the dermis of SKH-1 mice. Given the sparse and even contradictory existing information on the effect of UVB radiation on dermal fibroblasts’ viability, aim of this work was to unravel the crucial signaling pathways regulating the survival of UVB-treated human dermal fibroblasts. We found that UVB radiation immediately stimulates the phosphorylation of MAPK family members, as well as Akt, and is genotoxic leading to the delayed ATM-p53 axis activation. Akt phosphorylation after UVB radiation is EGFR-mediated and EGFR inhibition leads to a further decrease of viability, while the Akt activator SC79 rescues fibroblasts to an extent by a mechanism involving Nrf2 activation. The known Nrf2 activator sulforaphane also exerts a partial protective effect, although by acting in a distinct mechanism from SC79. On the other hand, inhibition of JNKs or of the ATM-p53 axis leads to a complete loss of viability after UVB irradiation. Interestingly, JNKs activation is necessary for p53 phosphorylation, while the ATM-p53 pathway is required for the long-term activation of JNKs and Akt, reassuring the protection from UVB. Although UVB radiation results in intense and prolonged increase of intracellular ROS levels, classical anti-oxidants, such as Trolox, are unable to affect Akt, JNKs, or p53 phosphorylation and to reverse the loss of fibroblasts’ viability. Collectively, here we provide evidence that the main viability-regulating UVB-triggered biochemical pathways act synergistically towards the protection of human dermal fibroblasts, with EGFR/Akt and Nrf2 serving as auxiliary anti-apoptotic machineries, while JNKs/ATM-p53 activation and interplay being overriding and indispensable for the perpetuation of cellular defense and the maintenance of cell viability.

## Introduction

The skin is the largest organ of the human body that, due to its outermost anatomical location, acts as the first defense against adverse physicochemical, biological and environmental insults, a major being solar ultraviolet (UV) radiation that can result in short-term sunburn (leading to erythema and edema) or to chronic sun damage provoking premature skin aging (the so-called photoaging) and even carcinogenesis [1–3]. While UVC (200–280 nm) is mainly absorbed by the atmosphere, UVA (315–400 nm) and UVB (280–315) are the UV fractions that actually reach the earth and consequently affect human and animal skin [4]. UVA comprises the main segment of terrestrial UV radiation and can penetrate the skin to a depth of ~1000 μm. UVB instead is mainly absorbed by the epidermal cornified layer that allows 5–10% of its photons to attain the papillary layer of the dermis, as it penetrates the skin to a depth of ~160–180 μm [5–8].

UV radiation-induced skin tissue impairment is the cumulative result of the UV-triggered genotoxicity and production of reactive oxygen species (ROS) [3]. While the effects of UVA are primarily oxidative in nature [2], UVB has a multifaceted and still ambiguous mode of action: UVB photons are much more energetic compared to UVA ones, with a stronger cytotoxic and genotoxic effect on the residing cells, as they are more capable of directly interacting with DNA and introducing DNA damage to skin cells [9, 10]; yet, UVB radiation can also interact with cellular chromophores that provoke oxidative stress, which in its turn may cause indirect DNA damage [11–14].

Given that UVB wavebands are mostly absorbed by the epidermis, current literature has primarily focused on the effect of UVB radiation on epidermal keratinocytes, having assessed several physiological parameters. More specifically, UVB radiation has been shown to induce an apoptotic cell death in keratinocytes [15], which is ROS-mediated [16]. This UVB-induced apoptosis has been shown to be significantly reduced in keratinocytes after the activation of the transcription factor NF-E2-related factor 2 (Nrf2) and the consequent reduction of ROS levels in transgenic mice in vivo [17, 18]. ERK1/2, JNKs, and p38 signaling pathways have been reported to be activated via ROS in human keratinocytes as a protective mechanism towards the ROS-mediated cell death provoked by UVB treatment [19, 20]. In addition, increased ERK and Akt activation have been demonstrated to enhance the survival of keratinocytes after exposure to UVB radiation by suppressing PTEN [21]. Furthermore, the key DNA damage response in UVB-treated keratinocytes has been reported to be the ataxia telangiectasia and Rad3-related (ATR)-Chk1 pathway [22], while UVB-triggered activation of p53 signaling has been mainly associated with the induction of apoptosis in these cells [23].

On the other hand, much less concern has been devoted to the cytotoxic effect of UVB radiation on the dermis and its major cell type, i.e., fibroblasts. Previous studies on UVB-irradiated dermal fibroblasts have thus far concentrated mostly on extracellular matrix and the induction of cellular senescence and photoaging [5, 24, 25], with sparse and equivocal information existing on the effect of UVB radiation on their viability. Since data reported are conflicting and even sometimes contradictory, due to species-specific and irradiation dose-dependent differences, here we aimed to unravel the crucial intracellular biochemical pathways activated after exposure to physiologically relevant UVB doses that ultimately determine the fate of irradiated dermal fibroblasts towards UVB-induced cell death or evasion from cytotoxicity.

## Materials and methods

### Chemical reagents

Primary antibodies specific to phospho-p38 (Thr180/Tyr182) MAPK, p38 MAPK, phospho-ATM (Ser1981), phospho-p53 (Ser15), phospho-Akt (Ser473), phospho-Akt (Thr308), total Akt, phospho-SAPK/JNK, SAPK/JNK, phospho-EGF receptor (EGFR) (Tyr1068), caspase-3 and cleaved caspase-3 (Asp175), were purchased from Cell Signaling Technology (Beverly, MA, USA). Phospho-ERK (Thr202/Tyr204) and panERK antibodies were supplied by BD Pharmingen (Bedford, MA, USA) and the pan-actin antibody by Neomarkers, Lab Vision (Fremont, CA, USA). Rabbit polyclonal antibody against Nrf2 and mouse monoclonal antibody p53 (DO-1) were purchased from Santa Cruz Biotechnology (Santa Cruz, CA, USA). Anti-phospho-H2A.X (Ser139) used in immunofluorescence experiments was purchased from Merck Millipore (Darmstadt, Germany), while anti-phospho-H2A.X (Ser139), clone JBW301 used in immunohistochemical analysis was from Sigma (St. Louis, MO, USA). Anti-α-tubulin clone DM1A was purchased from Sigma. Secondary anti-mouse and anti-rabbit HRP-conjugated IgG antibodies were purchased from Sigma and FITC-conjugated IgG was from Santa Cruz Biotechnology. EGF was from Sigma. The specific to p38 MAPK (SB203580), SAPK/JNKs (SP600125) and ERK (PD98059) inhibitors were obtained from Sigma, the highly specific inhibitor of Akt1/2/3 (MK2206) was purchased from TargetMol (Wellesley Hills, MA, USA) and the specific to ataxia telangiectasia mutated (ATM) kinase inhibitor KU55933 was kindly provided by KuDOS Pharmaceuticals (Cambridge, UK). The PDGF receptor (PDGFR) kinase inhibitor STI571 was supplied by Novartis AG (Basel, Switzerland), the VEGF receptor (VEGFR) and FGF receptor (FGFR) inhibitor SU5402, the EGFR kinase inhibitor tyrphostin AG1478 and the IGFI receptor (IGFIR) kinase inhibitor tyrphostin I-OMe-AG538 were from Calbiochem-Merck KGaA (Darmstadt, Germany), the EGFR inhibitor PD153035 was from MedChemExpress Co., Ltd (Monmouth Junction, NJ, USA) and the TGF-β type I receptor (TGFβR1) kinase inhibitor SB431542 was purchased from Sigma. The anti-oxidant 6-hydroxy-2,5,7,8-tetramethylchroman-2-carboxylic acid (Trolox), the growth factor receptor antagonist suramin sodium salt, and the Nrf2 activator L-sulforaphane were supplied by Sigma, while the PI3K/Akt activator SC79 was purchased from Tocris Bioscience (Bristol, UK). Primer sequences, scramble, and siRNA sequences for p53 and Nrf2 were purchased from Eurofins Genomics (Ebersberg, Germany). SignalSilence^®^ Akt siRNA I targeting Akt1 and Akt2 was supplied by Cell Signaling Technology.

### Cells and cell culture conditions

Neonatal foreskin fibroblasts (AG01523c) used in this study were from the Coriell Institute for Medical Research (Camden, NJ, USA), primary human dermal fibroblasts from an adult donor were from a preexisting cell bank of the Laboratory of Cell Proliferation and Ageing [26], while MDAH041 fibroblasts from a Li-Fraumeni patient were a generous gift of Dr. M. Agarwal. All cells were regularly tested for mycoplasma contamination. Normal and MDAH041 fibroblasts were grown in Dulbecco’s Modified Eagle’s Medium (DMEM) supplemented with penicillin (100 U/mL)/streptomycin (100 μg/mL) (all from Biochrom AG, Berlin, Germany) and 15% and 10% (v/v) fetal bovine serum (FBS, from Gibco BRL, Invitrogen, Paisley, UK), respectively. Cell cultures were incubated at 37 °C in a humidified 5%-CO_2_-in-air atmosphere and subcultured twice a week at a 1:2 split ratio using a trypsin-citrate (0.25–0.3% w/v) solution.

### UVB irradiation

Cells were cultured until 90% of confluence and culture medium was replaced by serum-free and phenol red-free medium. For UVB irradiation, culture dishes with the cells were placed in the center of a UV box and irradiated under a UVB lamp (Sankyo Denki Co., Kanagawa, Japan) which had an emission spectrum of 280–360 nm and a peak at 306 nm. Intensity of the applied UVB light in mW/cm^2^ was measured using a VarioControl UV-meter (Waldmann Medizintechnik GmbH, Villingen-Schwenningen, Germany), while total energy of the irradiation dose hitting the sample in mJ/cm^2^ was calculated by multiplying intensity value with the duration of exposure in sec. UVB irradiation doses ranged from 35 to 3100 mJ/cm^2^. After irradiation, cells were incubated at 37 °C in a humidified atmosphere containing 5% CO_2_ for the indicated time periods. Cells that were not exposed to UVB radiation served as the untreated control.

Three months-old female SKH-1 hairless mice were exposed to UVB irradiation doses of 140 and 350 mJ/cm^2^ before sacrifice and surgical removal of the dorsal skin at the designated time-points post-irradiation and preparation of formalin-fixed, paraffin-embedded (FFPE) tissue sections for immunohistochemical analysis. Mice that were not exposed to UVB irradiation served as the untreated control. Five animals were randomly allocated to each group. Experiments were performed under the approval of the ethical committee of the NCSR “Demokritos” Animal Facility (6879/19–12–2017).

### Estimation of cell viability

Cell viability was estimated using the Neutral Red assay, as previously described [27, 28]. In brief, cells were detached by trypsinization and resuspended in culture medium. Neutral Red (Biochrom AG) was added at a final concentration of 0.0075% (w/v) in the cell suspension and after a 15-min incubation at 37 °C, red-colored metabollicaly active cells were measured using a haemocytometer.

### Cell cycle analysis

Cell cycle analysis was performed by flow cytometry, as described previously [29, 30]. Adhering and floating cells were collected via trypsinization and centrifugation. Cell pellets were washed with phosphate-buffered saline (PBS), fixed with 50% (v/v) ice-cold ethanol, and stained with 50 μg/ml of propidium iodide (PI, Sigma), in the presence of 5 mM MgCl_2_ and 10 μg/ml RNAse A (Sigma). Samples were analyzed on a FACS Calibur flow cytometer (Becton-Dickinson, San Jose, CA, USA) and experimental results were processed with Modfit (Verity Software House, Topsham, ME, USA).

### Real-time reverse transcription polymerase chain reaction (real-time RT-PCR)

Gene expression analysis was performed by reverse transcription-quantitative polymerase chain reaction (RT-qPCR), as previously described [31–33]. Total RNA was extracted with Trizol reagent (Invitrogen), while RNA concentration and purity were determined using a Nanodrop ND-1000 spectrophotometer (Nanodrop Technologies, Wilmington, DE, USA). cDNA was synthesized from 0.5 μg of RNA to a total volume of 10 μl using the PrimeScript RT Reagent Kit (Takara, Tokyo, Japan), according to the manufacturer’s instructions. The qPCR reaction (20 μl) was performed with cDNA at a final dilution of 1:100 using the KAPA SYBR FAST qPCR kit (KAPA Biosystems, Boston, MA, USA). Experiments were conducted in a Mx3000P qPCR Systems Cycler and data analysis was performed with MxPro QPCR Software (Stratagene, La Jolla, CA, USA). The 2^−ΔΔCt^ method was applied to quantify mRNA levels [34] and glyceraldehyde-3-phosphate dehydrogenase (GAPDH) served as the reference gene. Sequences of highly purified salt-free primers used in this study are the following: HO-1 (forward primer: 5΄-GCC-CTT-CAG-CAT-CCT-CAG-TTC-C-3΄, reverse primer: 5΄-AGT-GGT-CAT-GGC-CGT-GTC-AAC-3΄), NQO1 (forward primer: 5΄-ATG-GGA-GAC-AGC-CTC-TTA-CTT-GC-3΄, reverse primer: 5΄-AAC-CAC-CAG-TGC-CAG-TCA-GC-3΄), Bcl-2 (forward primer: 5΄-GCA-TGC-GGC-CTC-TGT-TTG-ATT-TCT-3΄, reverse primer: 5΄-AGG-CAT-GTT-GAC-TTC-ACT-TGT-GGC-3΄), Bax (forward primer: 5΄-CTC-ACC-GCC-TCA-CTC-ACC-ATC-3΄, reverse primer: 5΄-CTC-AAG-ACC-ACT-CTT-CCC-CAC-AC-3΄), MDM2 (forward primer: 5΄-TGG-GCA-GCT-TGA-AGC-AGT-TG-3΄, reverse primer: 5΄-CAG-GCT-GCC-ATG-TGA-CCT-AAG-A-3΄) and GAPDH (forward primer: 5΄-GAG-TCC-ACT-GGC-GTC-TTC-3΄, reverse primer: 5΄-GCA-TTG-CTG-ATG-ATC-TTG-AGG-3΄).

### Western blot analysis

Western blot analysis was performed as described previously [26, 29, 35]. For total protein extraction, cells were washed twice with ice-cold Tris-buffered saline (TBS, 10 mM Tris-HCl pH 7.4, 150 mM NaCl) and scraped off in hot Laemmli sample buffer supplemented with protease- and phosphatase-inhibitor cocktails (Sigma). Cell lysates were stored at −30 °C until use. SDS-PAGE was carried out in Bis-Tris polyacrylamide gels and proteins were transferred to Amersham Hybond P 0.45 PVDF blotting membranes (GE Healthcare, Buckinghamshire, UK). Membranes were blocked with 5% (w/v) non-fat milk in TBS, 0.05% Tween-20 (TBS-T) buffer for 1 h and incubated overnight with the appropriate primary antibodies at 4 °C. The next day membranes were washed three times with 5% (w/v) non-fat milk in TBS-T, incubated with either an anti-mouse or an anti-rabbit horseradish peroxidase-conjugated antibody for 1.5 h at room temperature, and washed again twice with 5% (w/v) non-fat milk in TBS-T and once with TBS-T. Immunoreactive bands were visualized using an enhanced ECL substrate kit (Immobilon Crescendo Western HRP substrate, Merck Millipore). Membranes were stripped and re-probed with antibodies against the respective non-phosphorylated forms of the proteins or with an anti-actin or anti-α-tubulin antibody to verify equal loading.

### Measurement of intracellular levels of reactive oxygen species (ROS)

Intracellular ROS levels were measured using the 2 ´,7 ´-dichlorfluorescein-diacetate (DCFH-DA) assay [31, 36]. Human skin fibroblasts were cultured in 96-well plates in DMEM supplemented with 15% FBS. When confluent, cells were pre-incubated with 10 μM of freshly diluted in serum-free DMEM DCFH-DA (Sigma) for 1 h at 37 °C, then exposed to the appropriate UVB irradiation dose and further incubated at 37 °C. Fluorescence was recorded at the selected time-points using a fluorescent microplate reader (Fluostar, Optima BMG LABTECH excitation wavelength: 485 nm, emission wavelength: 520 nm). ROS production was expressed as a % ratio of the untreated control.

### Immunofluorescence

For immunofluorescence experiments assessing H2A.X phosphorylation on Ser139 and Nrf2 localization, skin fibroblasts were cultured on glass coverslips before treatment and they were then fixed with 4% (w/v) formaldehyde in PBS. Labeling was performed as previously described [31, 37] using antibodies against phospho-H2A.X (Ser139) and Nrf2 and a FITC-conjugated IgG. Nuclear staining was performed with 2 μg/mL 4′,6-diamidino-2-phenylindole (DAPI) dihydrochloride (Sigma). Labeled cells were visualized using a Zeiss AxioPlan 2 microscope (Carl Zeiss, Jena, Germany) and a confocal laser scanning microscope (TCS SP8 multiphoton confocal microscope, Leica, Mannheim, Germany).

### Immunohistochemistry (IHC)

FFPE sections were deparaffinized (incubation at 60 °C for 30 min and one wash in xylene for 20 min) and rehydrated in gradually decreased ethanol solutions. The blocking of endogenous hydrogen peroxidase was performed using the UltraVision Hydrogen Peroxide Block included in the Ultravision Quanto Detection System HRP (Thermo Fisher Scientific, Cleveland, OH, USA) for 10 min at room temperature and in the dark. The next step after washes with TBS 1× was antigen retrieval, boiling tissue samples with citric acid 1× (pH 6.0) in a microwave (700 W) for 25 min or in a steamer for 40–50 min. In order to block non-specific epitopes, incubation with Ultra-V-Block buffer (included in the Ultravision Quanto Detection System HRP) was performed for 5 min at room temperature. For IHC experiments samples were incubated overnight at 4 °C with four different antibodies: a. anti-γH2A.X (Ser139), clone JBW301 diluted 1/1000 in TBS 1× supplemented with 1/20 goat serum (Thermo Fisher Scientific); b. anti-phospho-Akt (Ser473) (D9E) diluted 1/200 in TBS 1×; c. anti-cleaved caspase-3 (Asp175) diluted 1/100 in TBS 1×; d. anti-phospho-SAPK/JNK (Thr183/Tyr185) diluted in 1/100 in TBS 1× supplemented with 1/20 goat serum. The samples were incubated with Primary Antibody Amplifier Quanto (Thermo Fisher Scientific) for 10 min at room temperature and then with HRP Polymer Quanto (Thermo Fisher Scientific) for 15 min at room temperature in the dark. DAB Plus Chromogen diluted 1/100 in DAB Plus Substrate (Thermo Fisher Scientific) was used and the staining reaction was monitored under a light microscope for 50 s–1 min After washes with tap water, samples were counterstained with hematoxylin, dehydrated with gradually increased ethanol solutions and washed in xylene for 15 min. Mounting was performed using permanent mounting media (Agilent Technologies, Santa Clara, CA, USA). Slides were assessed in a randomized order by two blinded observers.

### siRNA transfection

Transfection of siRNA for p53, Nrf2, and Akt was performed as previously described [29, 30, 35]. In brief, human skin fibroblasts were plated in DMEM containing 15% (v/v) FBS until they reached 70% confluence. Then, cells were transfected with 50 nM of either a predesigned scramble (5′-UAAUGUAUUGGAACGCAUATT-3′), or the human p53 siRNA sequence (5′-CUACUUCCUGAAAACAACGTT-3′), or the human Nrf2 siRNA sequence (5΄-AAGAGUAUGAGCUGGAAAAACTT-3΄) or SignalSilence^®^ Akt siRNA I in serum-free OpitiMEM I medium using lipofectamine 2000 (Invitrogen). Five hours later, the transfection medium was replaced by culture medium supplemented with 15% (v/v) FBS and cells were incubated for another 48 h before any other treatment. Specificity of the selected human p53 and Nrf2 siRNA sequences has been confirmed previously [38, 39].

### Statistical analysis

Data presented are the means of at least three repeats ± standard deviation. Differences between groups of equal variance were considered significant when *p* < 0.05 (Student’s *t* test). Graphs were created using Microsoft Office Excel and GraphPad Prism version 5.0 (GraphPad Software, San Diego, CA, USA).

## Results

### UVB radiation induces apoptotic cell death in dermal fibroblasts in vitro and in vivo

**Fig. 1:**
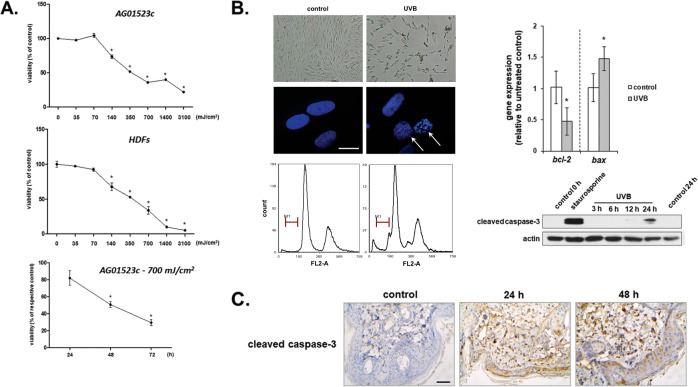
Exposure of human skin fibroblasts to UVB radiation leads them to a programmed cell death in vitro and in vivo. **A** AG01523c and primary human dermal fibroblasts from an adult donor (HDFs) were irradiated under a UVB lamp for durations corresponding to irradiation doses ranging from 35 to 3100 mJ/cm^2^. After incubation at 37 ^o^C for 72 h, cells were detached by trypsinization and red-colored metabollicaly active cells after staining with Neutral Red were measured using a haemocytometer. In addition, AG01523c fibroblasts were irradiated with a UVB dose of 700 mJ/cm^2^ before staining with Neutral Red and cell counting 24, 48, and 72 h post-irradiation. Untreated cells served as the reference sample for the estimation of cell viability. Representative graphs out of at least three similar ones from independent experiments are shown here. Asterisks denote statistically significant differences compared to the untreated control at the respective time-point (*p* < 0.05, Student’s *t* test). **B** Cells exposed to a 700 mJ/cm^2^ UVB irradiation dose were observed under a light or fluorescence [after staining with 2 μg/mL 4 ´,6-diamidino-2-phenylindole (DAPI)] optical microscope for the presence of typical apoptotic features; fixed with 50% (v/v) ice-cold ethanol and stained with PI before cell cycle analysis to reveal the incidence of characteristic sub-G1 peaks; subjected to RNA and protein extraction to assess *bcl-2* and *bax* mRNA levels by RT-qPCR and caspase-3 activation by western blot analysis, respectively. Representative microscopic images, histogram plots, relative gene expression graphs, and western blots of three independent experiments are shown. Arrows in the fluorescence microscopic pictures depict regions of condensed/fragmented chromatin in the DAPI stained nuclei (scale bar = 20 μm). M1 marker designates the sub-G1 peak corresponding to apoptotic cells with fragmented DNA preceding the characteristic G1 and G2/M peaks in the histogram plots of PI-stained cells. Asterisks denote statistically significant differences in the gene expression of UVB-exposed fibroblasts in comparison to the untreated control (*p* < 0.05, Student’s *t* test). Staurosporine was used as a known apoptosis inducer leading to caspase-3 cleavage, while western blot analysis against actin served as the loading control. **C** SKH-1 hairless mice were exposed to 350 mJ/cm^2^ of UVB radiation and the dorsal skin was surgically removed 24 and 48 h post-irradiation for the assessment of cleaved caspase-3 in situ expression in the skin sections after immunohistochemical staining. Pictures shown here are representative of three independent experiments. Scale bar = 50 μm.

Here we showed that UVB radiation is cytotoxic for a commercially available cell line of human skin fibroblasts in a dose- and time-dependent manner (Fig. 1A). This was also demonstrated using a series of primary human dermal fibroblasts (Fig. 1A and data not shown). It is noteworthy that cytotoxic UVB irradiation doses found in the current study are physiologically relevant, as they are in between the minimal erythema doses (MED) estimated for fair- and darker-skinned phototypes [40–42]. UVB-induced cell death was found to be apoptotic, as evidenced by typical traits, including DNA fragmentation, UVB dose-dependent up-regulation of the pro-apoptotic gene *bax* (Supplementary Fig. 1A) along with down-regulation of the anti-apoptotic gene *bcl-2*, as well as caspase-3 activation (Fig. 1B), in accordance to previous reports [4, 43–45]. UVB-induced apoptosis was also shown to occur in vivo, as we observed an increased proportion of cells expressing activated caspase-3 in the skin of irradiated SKH-1 hairless mice 24 and 48 h after exposure to UVB radiation (Fig. 1C). Interestingly, cleaved caspase-3-positive cells were found not only in the epidermis, but also in the dermis of UVB-irradiated skin, confirming the ability of UVB radiation to reach and damage the embedded fibroblasts.

### UVB-mediated Akt and JNKs activation is cytoprotective for human dermal fibroblasts

**Fig. 2:**
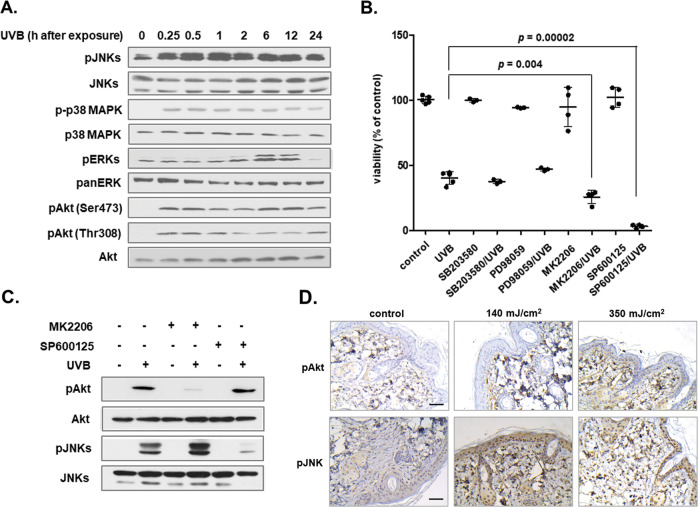
Akt and JNKs are the key players in the cellular response of human dermal fibroblasts towards UVB radiation. **A** Cells were irradiated with an irradiation dose of 700 mJ/cm^2^ and further incubated at 37 ^o^C for the designated time periods before total protein extraction, SDS-PAGE, protein transfer and western blot analysis with antibodies against the phosphorylated forms of JNKs, p38 MAPK, ERKs, and Akt. Representative blots of three independent experiments are shown here. Western blot analysis for the non-phosphorylated forms of the kinases was performed to verify equal loading. **B** Cells were pre-treated with the specific MAPKs and Akt inhibitors [SB203580 for p38 MAPK (10 μΜ); PD98059 for ERKs (25 μΜ), SP600125 for JNKs (5 μΜ), and MK2206 for Akt (1 μΜ)] for 1 h before UVB treatment with 700 mJ/cm^2^. Cells were further incubated at 37 ^o^C for 72 h before trypsinization, staining with Neutral Red and cell counting (*N* = 4). *p* for statistically significant differences in comparison to the respective sample without any inhibitor (Student’s *t* test) is presented in the graph. **C** Cell cultures were pre-incubated with 1 μΜ of the Akt inhibitor MK2206 and 5 μΜ of the JNKs inhibitor SP600125 for 1 h before exposure to 700 mJ/cm^2^ of UVB radiation, total protein extraction and western blot analysis for phospho-Akt at Ser473 and phospho-JNKs. The non-phosphorylated forms of the kinases served as loading controls. Western blot analyses were repeated three times and a representative experiment is presented here. **D** SKH-1 hairless mice were exposed to 140 and 350 mJ/cm^2^ of UVB radiation before sacrifice 1 h post-irradiation and removal of the dorsal skin. Non-irradiated (control) *vs*. irradiated tissue sections were stained using antibodies against the phosphorylated forms of Akt at Ser473 and of JNKs. Pictures of pAkt and pJNKs in situ expression shown here are representative of three independent experiments. Scale bar = 50 μm.

Exposure to UVB radiation led to a rapid and sustained phosphorylation of p38 MAPK and JNKs and a delayed activation of ERKs in human dermal fibroblasts (Fig. 2A), consistent with the activation of MAPK family members reported earlier for these cells [46, 47]. In addition, we found that UVB treatment leads to an immediate and long-lasting phosphorylation of Akt at both Ser473 and Thr308 (Fig. 2A), in agreement with previous reports [48, 49]. Inhibition of each one of the UVB-activated kinases shown here using their selective pharmacological inhibitors (i.e., SB203580 for p38 MAPK, PD98059 for ERKs, MK2206 for Akt and SP600125 for JNKs) revealed that Akt and JNKs play a key role in cytoprotection. JNKs were found to be more pivotal for survival than Akt, since pre-treatment with SP600125 rendered cells entirely defenseless towards UVB radiation, whereas pre-treatment with MK2206 sensitized UVB-exposed fibroblasts, but still allowed the incidence of viable cells after irradiation (Fig. 2B). Akt and JNKs pathways were not found to be mutually reliant, since Akt inhibition did not block UVB-mediated JNKs phosphorylation and vice-versa (Fig. 2C). Interestingly, UVB-induced Akt and JNKs activation was confirmed in vivo by the observation of positive for phospho-Akt and phospho-JNKs cells in the dermis of SKH-1 mice shortly after UVB irradiation (Fig. 2D).

### JNKs are implicated in the activation of the DNA damage response of dermal fibroblasts after UVB treatment

**Fig. 3:**
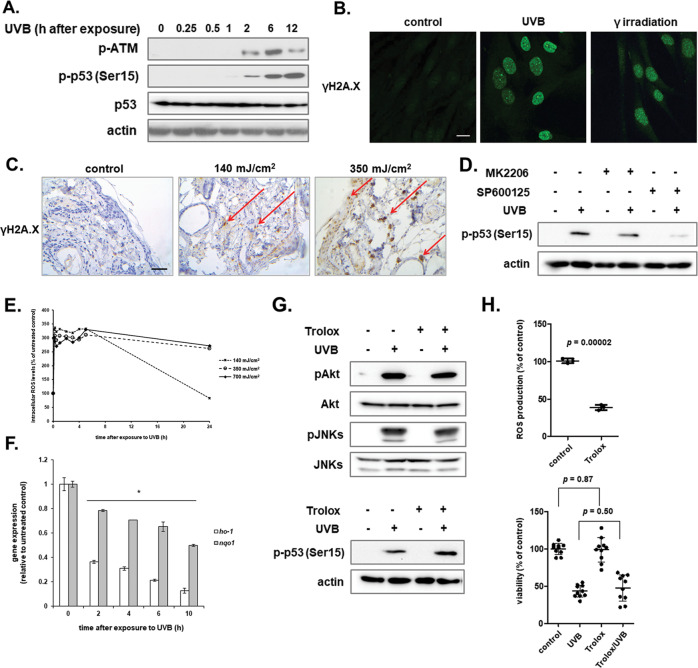
UVB radiation exerts a ROS-independent genotoxic effect on human dermal fibroblasts in vitro and in vivo. **A** Cells were exposed to 700 mJ/cm^2^ of UVB radiation before protein extraction at the designated time-points post-irradiation and western blot analysis for phopsho-ATM, phospho-p53, and total p53. Western blot analysis for actin was performed to verify equal loading. Experiments were repeated three times and representative blots are presented. **B** Skin fibroblasts were cultured on glass coverslips before UVB treatment with 700 mJ/cm^2^ and further incubated at 37^ o^C for 24 h. They were then fixed with 4% (w/v) formaldehyde followed by labeling using an antibody against phospho-H2A.X at Ser139 and a FITC-conjugated IgG. Labeled cells were visualized using a confocal laser scanning microscope. Cells exposed to 10 Gy of ionizing radiation served as the positive control. Representative images of three independent experiments are shown here. Scale bar = 20 μm. **C** SKH-1 hairless mice were exposed to 140 and 350 mJ/cm^2^ of UVB radiation before sacrifice and removal of dorsal skin 6 h post-irradiation. Non-irradiated (control) vs. irradiated tissue sections were subjected to immunohistochemical staining for the phosphorylated form of H2A.X at Ser139. Pictures shown here are representative of three independent experiments and arrows depict γH2A.X-positive cells in the epidermis and dermis of SKH-1 hairless mouse skin. Scale bar = 50 μm. **D** Cell cultures were pre-incubated with 1 μΜ of the Akt inhibitor MK2206 and 5 μΜ of the JNKs inhibitor SP600125 for 1 h before exposure to 700 mJ/cm^2^ of UVB radiation, protein extraction and western blot analysis for phopsho-p53 at Ser15. Western blot analysis using an anti-actin antibody was performed for the validation of the equal loading. A representative experiment of three similar ones is depicted here. **E** Human skin fibroblasts were cultured in 96-well plates. When confluent, cells were pre-incubated with 10 μM 2 ´,7 ´-dichlorfluorescein-diacetate (DCFH-DA) for 1 h at 37 ^o^C and then exposed to 140, 350, and 700 mJ/cm^2^ of UVB radiation. Intracellular levels of ROS were measured at the designated time-points post-irradiation by recording fluorescence (excitation wavelength: 485 nm, emission wavelength: 520 nm). ROS production was expressed as a % ratio of the untreated control. **F** Cells were exposed to an irradiation dose of 700 mJ/cm^2^ and further incubated at 37 ^o^C for 2, 4, 6, and 10 h before RNA extraction and RT-qPCR analysis using specific primers for HO-1 and NQO1. The 2^−ΔΔCt^ method was applied to quantify mRNA expression and glyceraldehyde-3-phosphate dehydrogenase (GAPDH) was used as the reference gene. A representative RT-qPCR analysis performed in triplicates is depicted here. The asterisk denotes statistically significant differences in comparison to the untreated control (*p* < 0.05, Student’s t-test). **G** Cells were pre-incubated with 20 μΜ of the known anti-oxidant Trolox before UVB treatment with 700 mJ/cm^2^ and total protein extraction at 1 and 12 h for western blot analysis for phospho-Akt (Ser473)/JNKs and phospho-p53, respectively. Representative images of three independent experiments with similar results are shown. The non-phosphorylated forms of the kinases, as well as actin served as the loading controls. **H** Cells pre-incubated with 20 μΜ Trolox overnight were treated with 10 μM DCFH-DA for 1 h at 37 ^o^C before measurement of intracellular ROS levels. ROS production was expressed as a % ratio of the untreated control (*N* = 3). In parallel, cells pre-treated with 20 μΜ Trolox overnight were exposed to 700 mJ/cm^2^ of UVB radiation. Cultures were further incubated at 37 ^o^C for 72 h before trypsinization and measurement of cell number after staining with Neutral Red using a haemocytometer (*N* = 5, conducted in duplicates). Differences in comparison to the untreated control are considered statistically significant when *p* < 0.05 (Student’s *t* test).

We demonstrated that UVB radiation is genotoxic for human dermal fibroblasts, resulting in the phosphorylation of ATM and its substrates p53 and Chk2 (Fig. 3A and data not shown), in the dose-dependent down-regulation of the p53 target gene *mdm2* (Supplementary Fig. 1B) in accordance to previous studies in keratinocytes [50, 51], as well as in the accumulation of γH2A.X foci in the nuclei of the cells (Fig. 3B). The phenomenon was verified in vivo, since γH2A.X-positive cells were observed not only in the epidermis, but also in the dermis of SKH-1 hairless mice, in accordance to previous data [10], once more confirming the ability of UVB radiation to reach the dermis, exerting its damaging effects on the embedded fibroblasts. Although ATR has been reported to be phosphorylated in UVB-exposed human keratinocytes [22, 52], we did not observe any ATR nor Chk1 phosphorylation in our cell model, also validated by the lack of effect of the highly selective and potent ATR inhibitor VE821 on UVB-induced loss of viability in human dermal fibroblasts (data not shown). Interestingly, JNKs activation was found to be required for the activation of the DNA damage response, given that treatment with SP600125 was shown for the first time to block UVB-induced p53 phosphorylation at Ser15 in UVB-treated human dermal fibroblasts (Fig. 3D). This finding is in accordance to the previously reported linkage of the JNK signaling pathway with the DNA damage response and with p53 stability and activity mainly regulating apoptosis in other cell and animal models [53, 54].

### UVB-induced loss of viability is not ROS-mediated in human dermal fibroblasts

Intracellular ROS levels were elevated immediately after UVB irradiation in human dermal fibroblasts and remained high for at least 24 h, depending on the irradiation dose used (Fig. 3E). The transcription factor Nrf2 is considered the master regulator of anti-oxidative responses [55] and there are some previous data on UVB-induced Nrf2 activation in keratinocytes and fibroblasts [56]. Interestingly, here UVB-induced oxidative stress did not result in the launching of an anti-oxidative response controlled by the transcription factor Nrf2, as shown by the down-regulation of its target genes HO-1 and NQO1 at the transcriptional level (Fig. 3F). This divergence may be ascribed to the different irradiation doses used between previous studies and ours. In the same vein, in our study, the use of a classical anti-oxidant (i.e., Trolox) had no effect on Akt, JNKs and p53 phosphorylation status nor on the UVB-induced viability loss of skin fibroblasts (Fig. 3G, H).

### EGFR activation participates in the mechanism leading to the protection of human dermal fibroblasts from UVB-induced cytotoxicity

**Fig. 4:**
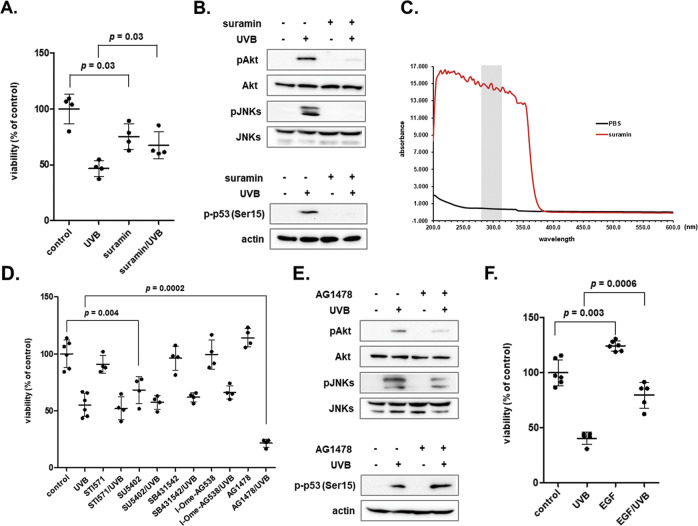
UVB-induced cellular responses are EGFR-Akt mediated in human skin fibroblasts. **A** Human dermal fibroblasts were pre-incubated with 100 μM suramin for 1 h before exposure to 700 mJ/cm^2^ of UVB radiation. Then, cells were further incubated at 37 ^o^C for 72 h and cell number was counted after staining with Neutral Red. A representative experiment out of three similar ones is shown. p for statistically significant differences in comparison to the respective samples in the absence of suramin (Student’s *t* test) is presented in the graph. **B** Cells were pre-treated with 100 μM suramin for 1 h before exposure to 700 mJ/cm^2^ of UVB radiation. Protein extracts after 1 and 12 h were subjected to western blot analysis for phospho-Akt (Ser473)/JNKs and phospho-p53, respectively. Representative blots of three independent experiments with similar results are depicted here. The non-phosphorylated forms of the kinases and actin were analyzed to verify equal loading. **C** A 100 μΜ suramin solution was analyzed spectrophotometrically in a range from 200 to 600 nm in order to assess its absorption spectrum. PBS served as the negative control. Gray shading marks the wavelength range corresponding to the UVB band (280–315 nm). **D** Cells were pre-incubated for 1 h with the growth factor receptor inhibitors [STI571 for PDGFR (2 μΜ); SU5402 for FGFR and VEGFR (20 μΜ); SB431542 for TGFβR1 (10 μΜ); I-OMe-AG538 for IGFIR (12 μΜ); AG1478 for EGFR (10 μΜ)] and then exposed to 700 mJ/cm^2^ UVB radiation. Cultures were further incubated at 37 ^o^C for 72 h, cells were detached by trypsinization, stained with Neutral Red and counted in a haemocytometer. *p* for statistically significant differences in comparison to the respective samples with no inhibitor (Student’s *t* test) is provided. **E** Cells were incubated with 10 μΜ of the EGFR inhibitor AG1478 for 1 h, exposed to 700 mJ/cm^2^ of UVB radiation and further incubated at 37 ^o^C for 1 or 12 h before protein extraction and western blot analysis for phospho-Akt (Ser473)/JNKs or phospho-p53, respectively. Representative blots of three independent experiments are shown here, while total Akt and JNKs, as well as actin were analyzed to validate equal loading. **F** Cell cultures were treated with 100 ng/ml EGF, exposed to 700 mJ/cm^2^ of UVB radiation and further incubated at 37 ^o^C for 72 h. Neutral Red-positive cells were counted in a haemocytometer. A representative graph from three independent similar experiments is presented. p for statistically significant differences in comparison to the respective samples in the absence of the growth factor (Student’s *t* test) is shown.

Even though UV radiation can directly impair tissue and cellular components, it has been well established that many of its effects are mediated by growth factors and cytokines that trigger intracellular signal transduction pathways [57–59]. In an attempt to elucidate the initial signal leading to UVB-mediated biochemical pathways’ activation in human dermal fibroblasts, we used the general growth factor receptor antagonist suramin [60]. Suramin was found to be protective against UVB cytotoxicity in our cell model (Fig. 4A), in agreement with its reported cytoprotective role in other cell types under stressful conditions [61, 62]. Pre-treatment with suramin totally abrogated Akt, JNKs and p53 phosphorylation in UVB-exposed human dermal fibroblasts (Fig. 4B); however, recording of suramin’s absorption spectrum revealed here for the first time that this effect is attributed to the ability of suramin to act as a UV shield (Fig. 4C). Thus, we followed by using the specific inhibitors of selected growth factor receptors, i.e., STI571 for PDGFR, SU5402 for FGFR and VEGFR, SB431542 for TGFβR1, I-OMe-AG538 for IGFIR and AG1478 for EGFR and deduced that only EGFR activation is necessary for human skin fibroblasts’ survival towards UVB radiation, as its inhibition further decreased UVB-induced cytotoxicity (Fig. 4D). This finding was also verified using a second EGFR inhibitor, PD153035 (Supplementary Fig. 2). EGFR was indeed rapidly phosphorylated by UVB treatment (Supplementary Fig. 2) and its inhibition by AG1478 abolished UVB-induced Akt phosphorylation, but did not affect JNKs and p53 activation (Fig. 4E). In accordance to the higher vulnerability of UVB-exposed cells after EGFR inhibition, exogenous supply of EGF restored survival of irradiated human dermal fibroblasts to an extent (Fig. 4F).

### The Akt activator SC79 protects human dermal fibroblasts from the UVB-mediated cytotoxic effect

**Fig. 5:**
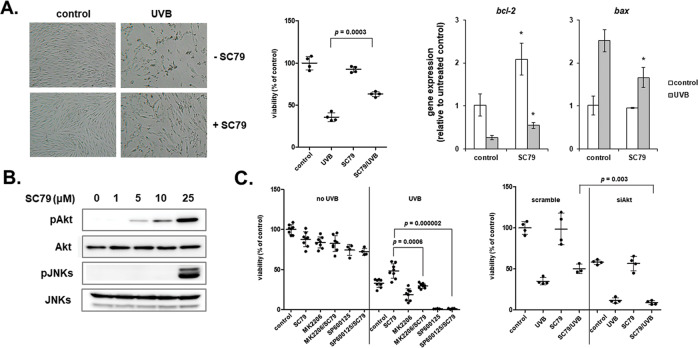
The known Akt activator SC79 protects human dermal fibroblasts from the cytotoxic effect of UVB radiation. **A** Cells were pre-treated with 25 μΜ SC79 for 1 h before their exposure to 700 mJ/cm^2^ UVB. After a further incubation at 37 ^o^C for 72 h, cells were observed under the microscope or detached by trypsinization and counted after staining with Neutral Red. In addition, RT-qPCR analysis for *bcl-2* and *bax* expression was performed in extracted RNA. Representative microscopic pictures are shown, while numerical values corresponding to the columns of the graphs are means ± standard deviations of three independent experiments conducted in triplicates. *p* for statistically significant differences in comparison to the respective sample without SC79 (Student’s *t* test) is presented. In the graphs of gene expression analysis, asterisks denote statistically significant differences in comparison to the respective sample without SC79 (*p* < 0.05, Student’s *t* test). **B** Cells were treated with SC79 at different concentrations (0, 1, 5, 10, and 25 μΜ) before total protein extraction at 1 h post-treatment and western blot analysis for the phosphorylated and non-phosphorylated forms of Akt and JNKs. Representative blots from three similar experiments are shown. **C** Cells were pre-treated with 1 μΜ of the Akt inhibitor MK2206 and 5 μM of the JNKs inhibitor SP600125 for 1 h or transfected with 50 nM scramble and SignalSilence^®^ Akt siRNA I sequences, before adding 25 μΜ SC79 and treating the cells with 700 mJ/cm^2^ of UVB radiation. Cell number was counted after 72 h of further incubation at 37 ^o^C and staining with Neutral Red. *p* for statistically significant differences in comparison to SC79/UVB-treated cells in the absence of inhibitor or Akt siRNA (Student’s *t* test) is presented.

We next used the Akt-specific activator SC79 that has been previously shown to protect retinal pigment epithelium cells from UVB and UVA2 radiation via Akt-Nrf2 signaling [63]. We demonstrated that SC79 is cytoprotective for UVB-exposed human dermal fibroblasts (Fig. 5A) by activating Akt as expected and - as shown here for the first time - by also activating JNKs (Fig. 5B). SC79-mediated protection against UVB radiation was lost in Akt-deficient cells or after inhibition of Akt activation by MK2206 (Fig. 5C), in agreement with previously reported data for retinal pigment epithelium cells [63]. Notably, SC79 protective effect against UVB radiation was also abrogated in human dermal fibroblasts after JNKs’ inhibition by SP600125 (Fig. 5C).

**Fig. 6:**
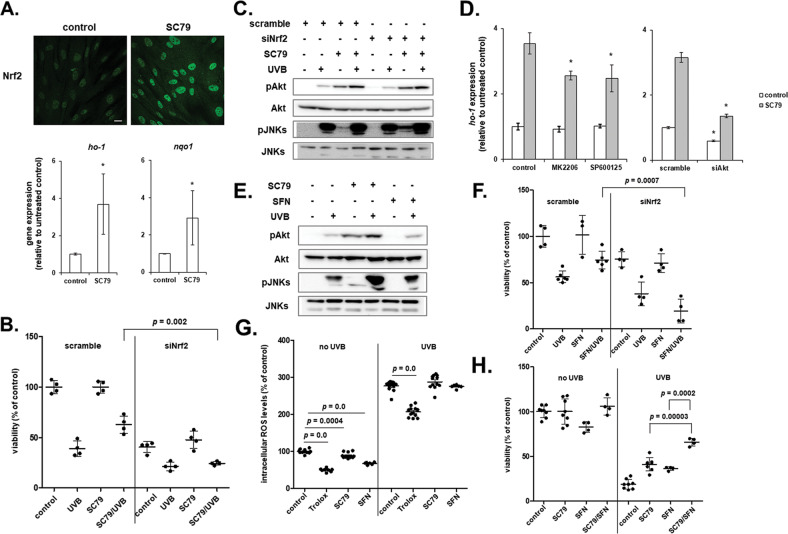
The Akt activator SC79 and the Nrf2 inducer sulforaphane are photoprotective for human dermal fibroblasts through Nrf2 activation, but without the implication of ROS scavenging. **A** Skin fibroblasts were cultured on glass coverslips before treatment with 25 μM of SC79 and they were then fixed with 4% (w/v) formaldehyde in PBS. Labeling was performed using an antibody against Nrf2 and a FITC-conjugated IgG. Labeled cells were visualized using a confocal laser scanning microscope (Scale bar = 20 μm). In addition, RNA was extracted and RT-qPCR analysis was performed to estimate *ho-1* and *nqo1* mRNA levels. Representative microscopic pictures and representative graphs of means ± standard deviations from three independent experiments conducted in triplicates are depicted here. Asterisks show statistically significant differences compared to the untreated control (*p* < 0.05, Student’s t-test). **B** Cells were transfected with 50 nM of either a scramble or an siRNA sequence targeting Nrf2, treated with 25 μΜ of SC79 for 1 h and then exposed to 700 mJ/cm^2^. Cell number was measured after 72 h in Neutral Red-stained cells. A representative experiment is presented out of three independent experiments. p for statistically significant difference in comparison to scramble SC79/UVB-treated cells (Student’s t-test) is provided. **C** Dermal fibroblasts were transfected with 50 nM of either scramble or Nrf2 siRNA, treated with 25 μΜ of SC79 for 1 h and then exposed to 700 mJ/cm^2^ UVB. Total protein extraction was performed 1 h post-irradiation followed by western blot analysis for phospho-Akt (Ser473) and phospho-JNKs. Representative blots of three independent experiments are shown. Western blot analysis of the non-phosphorylated forms of the kinases was done to evidence equal loading. **D** Cells were pre-treated with 1 μΜ of the Akt inhibitor MK2206 and 5 μM of the JNKs inhibitor SP600125 for 1 h or transfected with 50 nM scramble and SignalSilence^®^ Akt siRNA I sequences, before the addition of 25 μΜ SC79 for another 1 h. RT-qPCR analysis for *ho-1* gene expression was performed in the extracted RNA samples. A representative experiment from three similar ones is presented. Asterisks denote statistically significant differences in comparison to the respective samples without any inhibitor or siRNA (*p* < 0.05, Student’s *t* test). **E** Cells were treated with 25 μM SC79 or 10 μΜ sulforaphane (SFN) for 1 h and then exposed to an irradiation dose of 700 mJ/cm^2^ UVB before protein extraction and western blot analysis for phospho-Akt (Ser473) and phospho-JNKs, as well as their non-phosphorylated forms. Representative blots of three independent experiments are shown. **F** Cells were transfected with 50 nM of a scramble or a Nrf2 siRNA sequence, treated with 10 μΜ of SFN for 1 h and then exposed to 700 mJ/cm^2^. Cell number was measured after 72 h in Neutral Red-stained cells. A representative experiment out of three similar ones is presented. *p* for statistically significant difference in comparison to scramble SFN/UVB-treated cells (Student’s *t* test) is shown. **G** Cells were cultured in 96-well plates and treated with 20 μΜ Trolox, 25 μΜ SC79, or 10 μΜ SFN overnight. They were then incubated with 10 μM 2 ´,7 ´-dichlorfluorescein-diacetate (DCFH-DA) for 1 h at 37 ^o^C before their exposure to 700 mJ/cm^2^ of UVB radiation. Intracellular levels of ROS were estimated by recording fluorescence (excitation wavelength: 485 nm, emission wavelength: 520 nm). ROS production was expressed as a % ratio of the untreated control. Experiment was repeated three independent times and one representative graph is depicted. *p* for statistically significant differences compared to the respective sample without Trolox, SC79 or SFN (Student’s *t* test) is presented. **H** Cells were treated with 25 μΜ SC79, 10 μΜ SFN or both for 1 h and then exposed to 700 mJ/cm^2^. Cell number was measured after 72 h in Neutral Red-stained cells. A representative graph of three independent experiments is shown here. *p* for statistically significant difference in comparison to the UVB treated cells in the presence of SC79 or SFN alone (Student’s *t* test) is presented.

Despite the ROS-independent UVB-induced cytotoxicity shown here for human dermal fibroblasts, SC79 was found to activate Nrf2, as demonstrated by its translocation to the nucleus and by the up-regulation of its downstream target genes *ho-1* and *nqo1* (Fig. 6A), suggesting an implication of Nrf2 in SC79-conferred cytoprotection of human dermal fibroblasts against UVB radiation. Of note, Nrf2 knocking-down itself was found to sensitize human dermal fibroblasts to UVB exposure and this was not reversed by the presence of SC79 (Fig. 6B). As shown in Fig. 6C, SC79 acted by enhancing the intrinsic response of human skin fibroblasts towards UVB radiation, since it led to a more intense phosphorylation of the already increased by UVB treatment phosphorylated levels of both Akt and JNKs. Nrf2 activation seemed to lie downstream of the activation of both kinases, as this response remained unaltered by the loss-of-expression of Nrf2 (Fig. 6C). This finding was further validated after the inhibition of Akt and JNKs and the siRNA-mediated knocking-down of Akt that all attenuated SC79-induced *ho-1* and *nqo1* up-regulation in human skin fibroblasts (Fig. 6D and Supplementary Fig. 3A, respectively). To shed more light on the role of Nrf2 in UVB protection, we also used the known Nrf2 activator sulforaphane, which has been reported to possess photoprotective abilities in the skin [64]. We demonstrated that sulforaphane enhanced *ho-1* transcription, but resulted in no phosphorylation of Akt nor JNKs in human skin fibroblasts (Fig. 6E and Supplementary Fig. 3B), consistent with its known mode of action through the modification of Keap1 cysteines leading to the dissociation of the Keap1-Nrf2 complex [65]. Nrf2 knocking-down abolished its UVB protective effect (Fig. 6F). Given the overriding role of Nrf2 in ROS detoxification, we assessed the anti-oxidative capacity of both UVB-protectants, SC79 and sulforaphane, in our cell model. As shown in Fig. 6G, SC79 and sulforaphane protected human skin fibroblasts from UVB radiation by a mechanism that does not involve ROS scavenging, since only Trolox was found to lower UVB-induced intracellular ROS levels (which did not though confer any protective effect, as shown in Fig. 3H). Although they shared Nrf2 as a common molecular target, SC79 and sulforaphane seemed to act by distinct mechanisms, ultimately confirmed by their synergistic protective effect against UVB cytotoxicity (Fig. 6H).

### Activation of the ATM-p53 axis is indispensable for the cytoprotection of human skin fibroblasts against UVB radiation

**Fig. 7:**
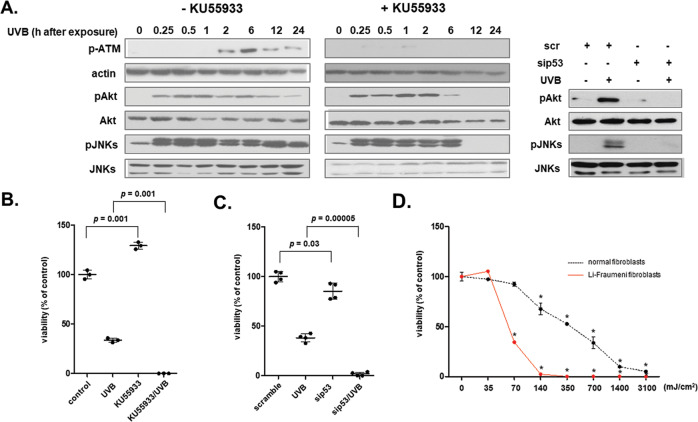
A functional DNA damage response is indispensable for the activation of human dermal fibroblasts’ defensive mechanisms towards UVB-induced cytotoxicity. **A** Cells were pre-incubated with 5 μM of the specific ATM inhibitor KU55933 for 1 h, exposed to 700 mJ/cm^2^ of UVB radiation and further incubated at 37 ^o^C for the designated time periods before total protein extraction and western blot analysis using antibodies against phospho-ATM at Ser1981, phospho-Akt at Ser473 and phospho-JNKs. Additionally, cells were transfected with 50 nM of either a predesigned scramble or the human p53 siRNA sequence before their exposure to 700 mJ/cm^2^ of UVB radiation, protein extraction 12 h post-irradiation and western blot analysis for phospho-Akt at Ser473 and phospho-JNKs. The non-phosphorylated forms of Akt and JNKs, as well as actin were analyzed in order to verify equal loading. Representative blots of three independent experiments are shown. Cells were pre-incubated with 5 μM of the specific ATM inhibitor KU55933 for 1 h (**B**) or transfected with 50 nM of either scramble or p53 siRNA (**C**), exposed to 700 mJ/cm^2^ of UVB radiation and further incubated at 37 ^o^C for 72 h before staining with Neutral Red and cell counting. Graphs are representative of three independent experiments and p for statistically significant differences in comparison to the respective sample without the inhibitor (**B**) or to the respective scramble (**C**) is presented. **D** MDAH041 fibroblasts from a Li-Fraumeni patient were irradiated with UVB irradiation doses ranging from 35 to 3100 mJ/cm^2^. After incubation at 37 ^o^C for 72 h, cells were detached by trypsinization, stained with Neutral Red, and measured using a haemocytometer. Untreated cells served as the reference sample for the estimation of cell viability. A representative graph out of three similar ones from independent experiments is shown here. Asterisks denote statistically significant differences in comparison to the respective untreated control (*p* < 0.05, Student’s *t* test).

Having shown the requirement of active JNKs for UVB-induced p53 phosphorylation in Fig. 3D, we next investigated the putative inverse association between the UVB-induced DNA damage response and the activation of the cytoprotective kinases Akt and JNKs. We showed that ATM inhibition, using its specific inhibitor KU55933, has no effect on Akt and JNKs phosphorylation at early time-points (up to 6 h post-UVB exposure); however, at later time-points, KU55933 completely blocked the activation of both kinases (Fig. 7A). Similarly, elimination of Akt and JNKs phosphorylation 12 h post-UVB exposure was also observed after p53 loss-of-expression using a specific siRNA sequence (Fig. 7A). These findings suggest that UVB-mediated activation of the ATM-p53 axis is essential for the generation of a second wave of Akt and JNKs’ activation, following initial phosphorylation that may explain their prolonged activation for several hours shown in Fig. 2A. In addition, any impairment of the DNA damage response (e.g., by ATM inhibition with KU55933 or by siRNA-mediated p53 knocking-down) was deleterious for UVB-exposed cells, leading to an absolute loss of cell viability (Fig. 7B, C and Supplementary Fig. 4). In line with this observation, fibroblasts derived from a Li-Fraumeni patient [66] owning a frameshift mutation in one p53 allele [67] demonstrated a much higher vulnerability towards UVB radiation compared to normal dermal fibroblasts (Fig. 7D). All the above evidence that the ATM-p53 axis is the most crucial anti-apoptotic UVB-triggered biochemical pathway for human dermal fibroblasts.

## Discussion

The skin is ubiquitously and inevitably exposed to the sun-emitted UVB and UVA radiation. UV radiation elicits a constellation of pathological events, the severity of which depends on the wavelength: the shorter UVB waveband is usually more toxic, but largely absorbed by the epidermis [3]; the longer UVA waveband, on the other hand, is much more penetrating, but less energetic and harmful. Given the thus far limited and conflicting information on the cellular mechanisms underlying UVB-triggered toxic effects on human dermal fibroblasts, here we aimed to elucidate the key biochemical pathways regulating survival of these cells after their exposure to UVB radiation.

We showed that UVB radiation leads to the apoptotic cell death of human dermal fibroblasts in accordance with a recently published work showing that UVB exposure may severely deplete papillary fibroblasts in human skin [68]. Based on the classification of the mechanisms of UV-induced apoptosis as immediate or delayed, the kinetics of caspase-3 activation (cleaved not before 24 h post-UVB exposure, following the activation of the ATM-p53 axis) found here points rather to a delayed than an immediate apoptosis, congruent with the short wavelength of UVB [2, 3, 69]. Akt and JNKs were revealed here to be crucial biochemical pathways for the regulation of cell viability in human dermal fibroblasts exposed to UVB radiation. In particular, UVB-induced Akt activation of human dermal fibroblasts was demonstrated to be mediated by EGFR. It is worth mentioning that while EGFR signaling has been shown to participate in the protection of human keratinocytes from UVB-induced apoptosis [70], this is the first time EGFR is shown to be implicated in the survival of human dermal fibroblasts after exposure to UVB radiation. Even though Akt has a well-known survival role in general that has been also shown previously in UVB-treated keratinocytes [21, 71–73], the anti-apoptotic role of JNKs in UVB-exposed dermal fibroblasts is more unconventional and in contradiction to the prominent function of the JNK pathway as a pro-apoptotic signal reported for UVB-treated keratinocytes [16] and UVC-irradiated fibroblasts [74]. However, in favor of our results, JNK inhibition by SP600125 has been demonstrated to augment UVB-induced apoptosis in retinal pigment epithelium cells [75].

Notably, UVB radiation induced ATM phosphorylation in human dermal fibroblasts in contrast to the ATR phosphorylation reported for UVB-exposed human keratinocytes [22], which may be attributed to the different cell type. Inability of human dermal fibroblasts to activate an Nrf2-mediated anti-oxidative response, despite the high UVB-induced ROS production, is consistent with the absence of ROS-induced Nrf2 activation reported in keratinocytes [76] and with the notion that ROS production may not necessarily be the main mediator of UVB signaling [58]. This is supported by previously published data: reduced nuclear Nrf2 and reduced cytosolic HO-1/NQO1 protein levels have been recently reported for UVB-treated human keratinocytes [77], while it has been shown that UVB radiation induces a Ca^2+^-dependent Nrf2 ubiquitination-mediated degradation in human dermal fibroblasts, accompanied by decreased HO-1 and NQO1 expression, similarly to our results [45]. In any case, the absence of Nrf2 activation and the down-regulation of the main ROS-elimination pathway observed in the current study could explicate the observed maintenance of high ROS levels for several hours post-UVB exposure in human dermal fibroblasts.

SC79 conferred a protective effect on UVB-treated skin fibroblasts dependent on Akt, as shown in other cell models under various stressful conditions [78–81]. Furthermore, SC79 was shown here for the first time to induce JNKs’ phosphorylation, as well. SC79-induced JNKs’ activation could contribute to its protective effect on UVB-exposed human dermal fibroblasts in accordance with anisomycin-induced JNK activation previously reported to attenuate UVB-induced apoptosis in retinal pigment epithelium cells [75]. Interestingly, despite the known main role of Nrf2 in the anti-oxidative response of the cells, protection conferred by SC79, as well as by sulforaphane (that both activated Nrf2) in UVB-exposed human dermal fibroblasts shown here did not rely on ROS scavenging. Nevertheless, recently reported evidence that Nrf2 may protect cells against DNA damage by mechanisms other than anti-oxidation that preserve genomic integrity [82] could justify the ROS-independent Nrf2-mediated protection against UVB geno- and cytotoxicity observed in our cell model. Moreover, given the established ability of Nrf2 to directly regulate the expression of matrisome-coding genes [83, 84], Nrf2 activation in human dermal fibroblasts could result in the production of an extracellular matrix that may protect cells from UVB-induced apoptosis.

The UVB-triggered DNA damage response manifested by the activation of the ATM-p53 axis was shown to be responsible for the long-lasting activation of Akt and JNKs in human dermal fibroblasts, thus establishing a positive feedback loop with p53 that enhanced and sustained Akt and JNKs activation and thus probably prolonged defense against UVB-induced cytotoxicity. Stress-induced p53 up-regulation has been reported to elicit JNK activation by preventing its rapid MAPK phosphatase-mediated dephosphorylation [85]. The evidenced here central role of the ATM-p53 axis could explain why a non-functional DNA damage response (due to ATM pharmacological inhibition, siRNA-mediated p53 knocking-down or p53 mutation ascribed to the Li-Fraumeni syndrome) rendered human dermal fibroblasts entirely defenseless towards UVB radiation, leading to a complete loss of cell viability. The dominance of this pathway and its utter requirement to warrant survival of human dermal fibroblasts towards UVB radiation was further reinforced by the dramatic effect of JNKs inhibition by SP600125 (that abolished UVB-induced p53 phosphorylation at Ser15). In contrast to the general notion that p53 plays a pro-apoptotic role competing Akt-driven anti-apoptotic signals in keratinocytes after exposure to UV radiation [86] and to previous data showing that ATM or p53 inhibition improves the viability of UVB-irradiated dermal cells from another species (i.e., mouse) [87], our findings in human dermal fibroblasts reveal an opposite role of the ATM-p53 axis that does not drive apoptosis, but instead co-operates with Akt and JNKs to secure survival in the UVB-exposed cells.

**Fig. 8:**
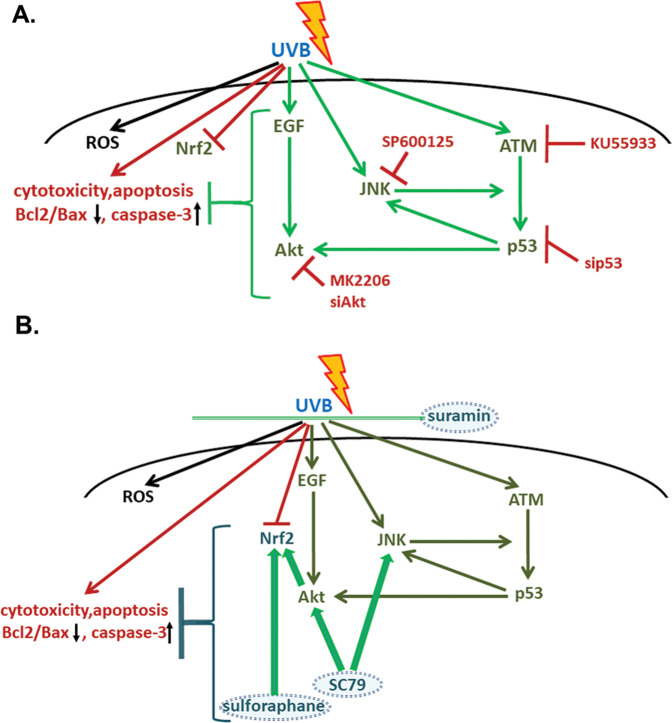
Proposed model of the UVB-triggered biochemical pathways in human dermal fibroblasts. **A** UVB-induced inherent cellular responses. UVB radiation leads human dermal fibroblasts to apoptosis in vitro and in vivo. UVB treatment induces in parallel the EGFR-Akt signaling pathway and the JNKs MAPKs which intervene with the phosphorylation of p53, part of the ROS-independent DNA damage response of the cells towards UVB genotoxicity. EGFR-Akt, JNKs, and ATM-p53 pathways seem to co-operate for the protection towards UVB radiation and the retention of a sufficient percentage of viable cells in the exposed population. This is evidenced by the increased loss of viability after Akt inhibition or siRNA-mediated knocking-down and the unmitigated cell death when directly (or indirectly through JNKs inhibition) impeding the activation of the ATM-p53 pathway. **B** Interplay of exogenously supplied photoprotective molecules with the intrinsic UVB-induced machinery to amplify the resistance of human skin fibroblasts against UVB radiation. Whereas the known anti-oxidant Trolox does not increase cell survival rate after UVB exposure, extra protection is conferred by other exogenously supplied molecules: suramin acts as a UV shield; EGF activates EGFR; SC79 enhances inherent cellular response (i.e., it activates further Akt and JNKs) and triggers Nrf2 activation downstream of Akt; sulforaphane induces Nrf2 activation independent of the Akt or JNKs signaling pathways. Nrf2-mediated protection by SC79 and sulforaphane is not based on ROS-scavenging and can be attributed to the transcription factor’s ability to safeguard genome integrity or to produce a protective extracellular matrix.

In conclusion, we showed here that the main biochemical pathways regulating cell viability after exposure of human dermal fibroblasts to UVB radiation are the Akt, the JNKs, and the ATM-p53 pathways (Fig. 8). In addition, while Nrf2 was not originally activated in UVB-exposed cells, its presence and functionality were shown to subserve protection against UVB-triggered cytotoxicity. Our data provide strong evidence on the cell type- and species-specificity, as well as on the dose dependence of UVB-induced cellular responses. What is mainly divergent in human dermal fibroblasts treated with a physiologically relevant UVB irradiation dose from data previously reported in keratinocytes or mouse dermal fibroblasts is that all implicated biochemical pathways found here act synergistically towards protection, with JNKs/ATM-p53 activation and interplay being indispensable for the perpetuation of cellular defense towards UVB radiation and the retainment of cell viability, while EGFR/Akt and Nrf2 being auxiliary anti-apoptotic machineries. Still, maintenance of damaged cells in the tissue may not always be beneficial, since accumulation of UVB-induced senescent dermal fibroblasts has been connected to skin photoaging and photocarcinogenesis [5]. Given the ever-expanding requirement for protection against photoaging and skin cancer due to the depletion of the ozone layer, the contemporary outdoor lifestyle, and the longer life expectancy, our data will hopefully contribute to the design of novel and more effective photoprotective strategies.

## Supplementary information

Supplementary Figures

Original Blots

Reproducibility checklist

## Data Availability

All data sets are included in this published article and its supplementary information. Additional data are available from the corresponding author on reasonable request.
