# Supplementary material for: Activation of the JNKs/ATM-p53 axis is indispensable for the cytoprotection of dermal fibroblasts exposed to UVB radiation
Source: Cell Death Dis. 2022 Jul 25;13(7):647. doi: 10.1038/s41419-022-05106-y (PMC9314411; doi:10.1038/s41419-022-05106-y)
Supplement: Supplementary file 1 — Supplementary Figures [file 41419_2022_5106_MOESM1_ESM.pdf]

Supplementary Figure 1

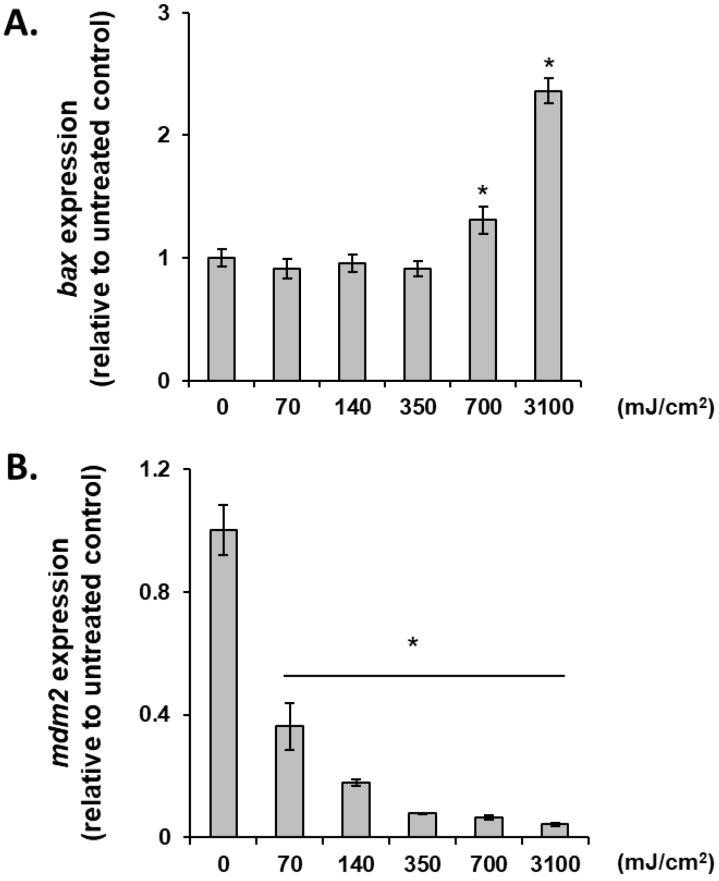

UVB-mediated regulation of p53 target genes is dose-dependent. Human skin fibroblasts were irradiated with irradiation doses ranging from 70 to 3100 mJ/cm^2 and further incubated at 37ºC before RNA extraction and RT-qPCR analysis using specific primers for Bax (A) and MDM2 (B). The $2^{-\Delta\Delta Ct}$ method was applied to quantify mRNA expression and glyceraldehyde-3-phosphate dehydrogenase (GAPDH) was used as the reference gene. Representative RT-qPCR analyses performed in triplicates are depicted here. Asterisks denote statistically significant differences in comparison to the untreated control ($p < 0.05$, Student's t-test).

Supplementary Figure 2

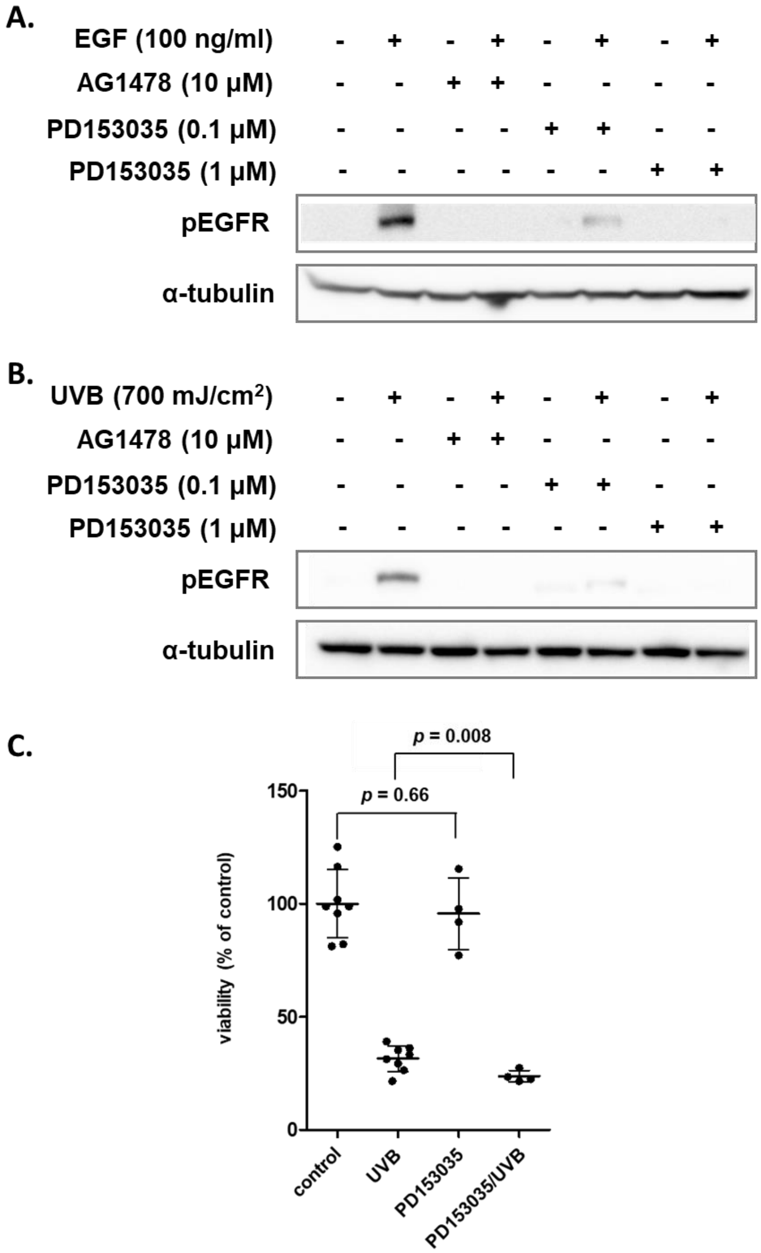

EGFR is rapidly phosphorylated after UVB treatment and is implicated in the cytoprotection of human dermal fibroblasts. A. Human dermal fibroblasts were pre-incubated or not with EGFR inhibitors AG1478 (10 μM) and PD153035 (0.1 and 1 μM) before treatment with 100 ng/ml EGF for 10 min, protein extraction and western blot analysis for phospho-EGFR at Tyr1068. Western blot analysis using an anti-α-tubulin antibody was performed for the validation of the equal loading. A representative experiment of three

similar ones is depicted here. B. Cells were pre-incubated or not with AG1478 (10 μM) and PD153035 (0.1 and 1 μM) before their exposure to a 700 mJ/cm^2 UVB dose and protein extraction 10 min post-irradiation. Western blot analysis was performed using antibodies against phospho-EGFR at Tyr1068 and α-tubulin. A representative experiment out of three repeats is shown. C. Cells were pre-incubated for 1 h with 0.1 μM of the EGFR inhibitor PD153035 and then exposed to 700 mJ/cm^2 of UVB radiation. Cultures were further incubated at 37°C for 72 h, cells were detached by trypsinization, stained with Neutral Red and counted in a haemocytometer. p for statistically significant differences in comparison to the respective samples with no inhibitor (Student's t-test) is provided.

Supplementary Figure 3

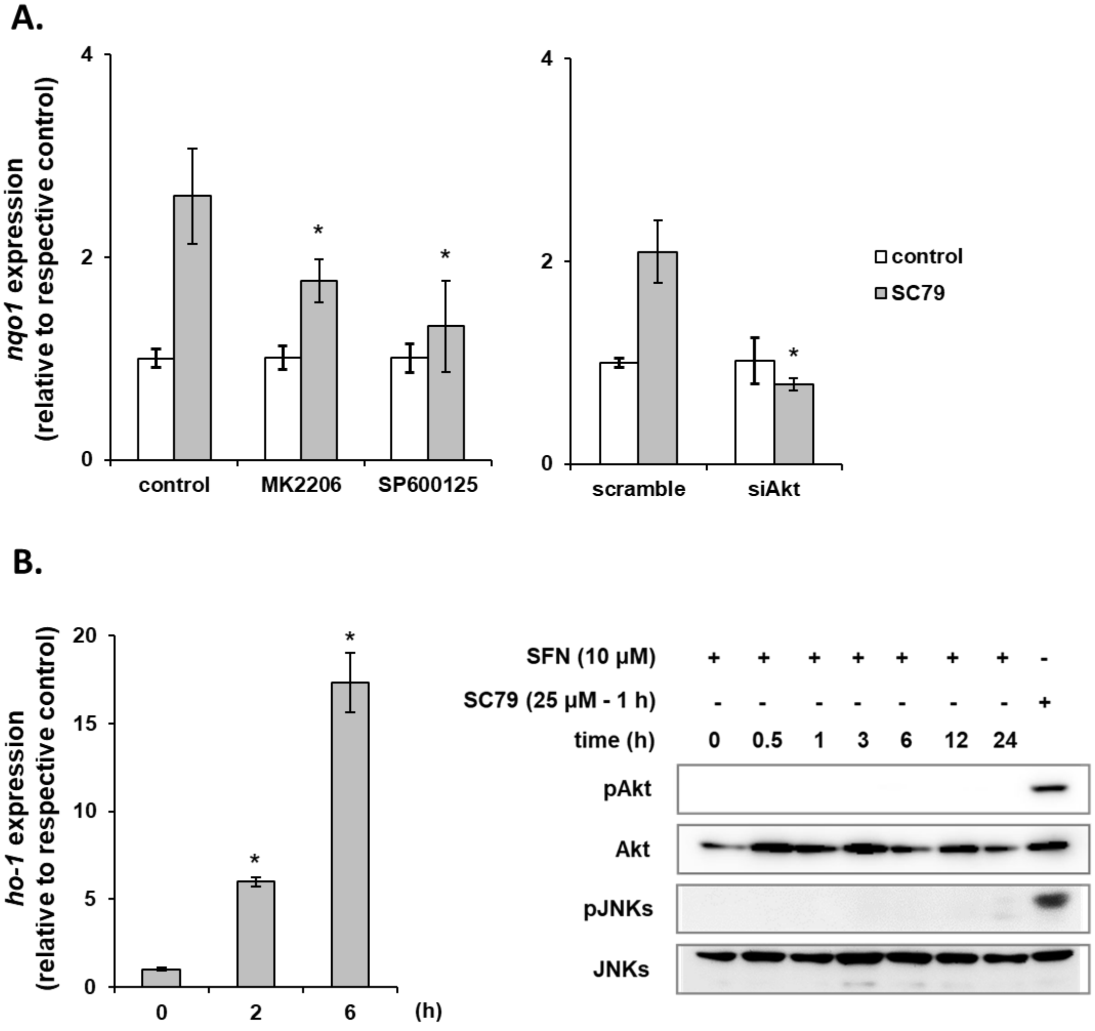

Both SC79 and sulforaphane are photoprotective for human dermal fibroblasts through Nrf2 activation, but sulforaphane does not lead to the phosphorylation of neither Akt nor JNKs. A. Human skin fibroblasts were pre-treated with 1 μM of the Akt inhibitor MK2206 and 5 μM of the JNKs inhibitor SP600125 for 1 h or transfected with 50 nM scramble and SignalSilence® Akt siRNA I sequences, before the addition of 25 μM SC79. RT-qPCR analysis for *nqo-1* gene expression was performed in the extracted RNA samples. A representative experiment out three similar ones is presented. Asterisks denote statistically significant differences in comparison to the respective samples without inhibitor or siRNA ($p < 0.05$, Student's t-test). B. Cells were treated with 10 μM of sulforaphane (SFN) for 0, 2 and 6 h before RNA

extraction and RT-qPCR analysis for *ho-1* gene expression. A representative graph of means ± standard deviations from three independent experiments is demonstrated. Asterisks show statistically significant differences compared to the untreated control ($p < 0.05$, Student's t-test). In addition, cells were treated with 10 μM of SFN for the designated time periods or with 25 μM of SC79 for 1 h and western blot analysis was performed in the protein extracts using antibodies against phospho-Akt (Ser473), phospho-JNKs and the non-phosphorylated forms of the kinases. Representative blots are shown here.

Supplementary Figure 4

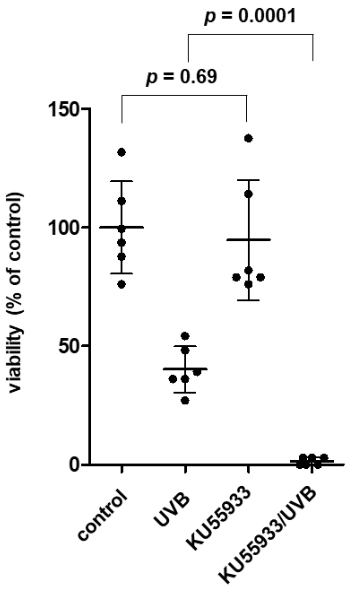

Impairment of the DNA damage response renders human dermal fibroblasts entirely defenseless towards UVB-induced cytotoxicity. Primary human dermal fibroblasts from an adult donor were pre-incubated with 5 µM of the specific ATM inhibitor KU55933 for 1 h, exposed to 700 mJ/cm^2 of UVB radiation and further incubated at 37°C for 72 h before staining with Neutral Red and cell counting. A representative graph from three independent experiments is depicted. p for differences in comparison to the respective sample without the inhibitor is presented.
