# Supplementary material for: Activation of the JNKs/ATM-p53 axis is indispensable for the cytoprotection of dermal fibroblasts exposed to UVB radiation
Source: Cell Death Dis. 2022 Jul 25;13(7):647. doi: 10.1038/s41419-022-05106-y (PMC9314411; doi:10.1038/s41419-022-05106-y)
Supplement: Supplementary file 2 — Original Blots [file 41419_2022_5106_MOESM2_ESM.pdf]

Original images of blots

Figure 1B

caspase-3

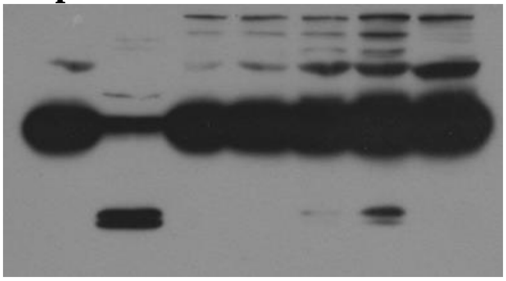

actin

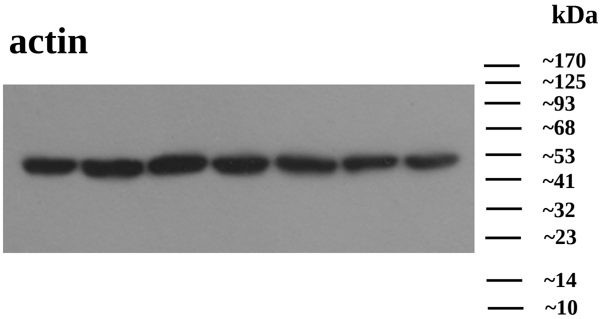

Figure 2A

pJNKs

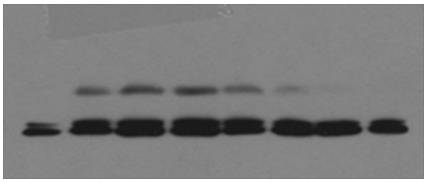

JNKs

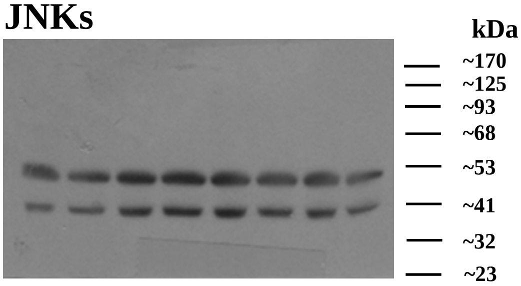

p-p38 MAPK

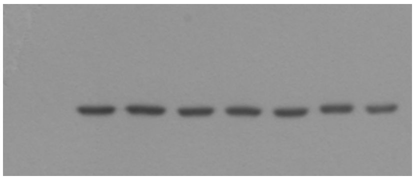

p38 MAPK

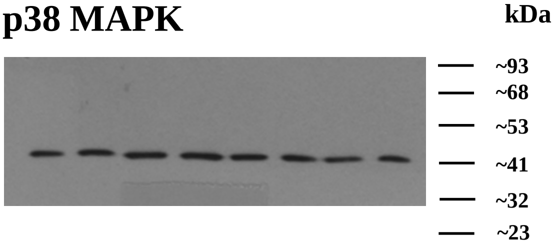

pERKs

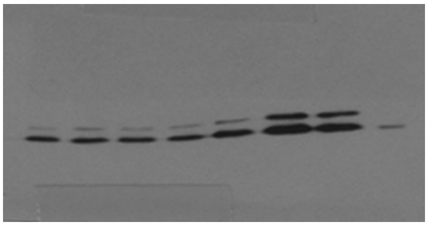

panERK

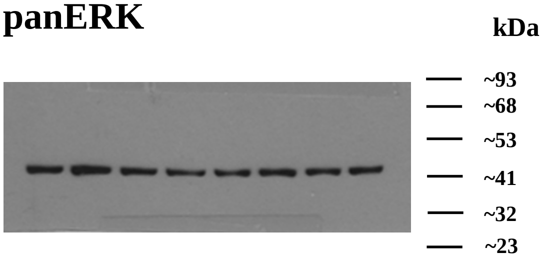

pAkt (Ser 473)

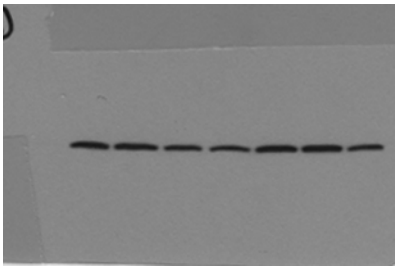

pAkt (Thr308)

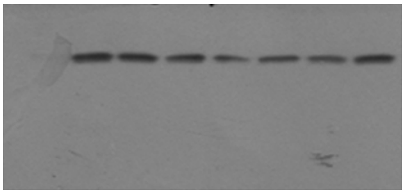

Akt

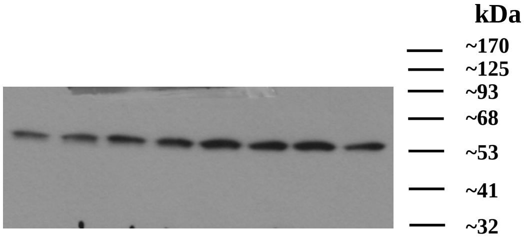

Figure 2C

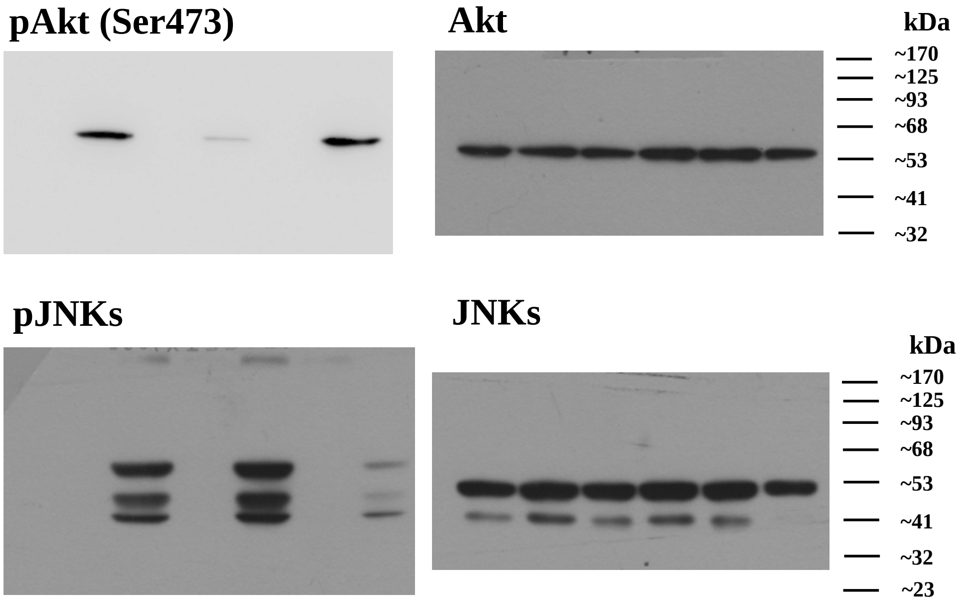

Figure 3A

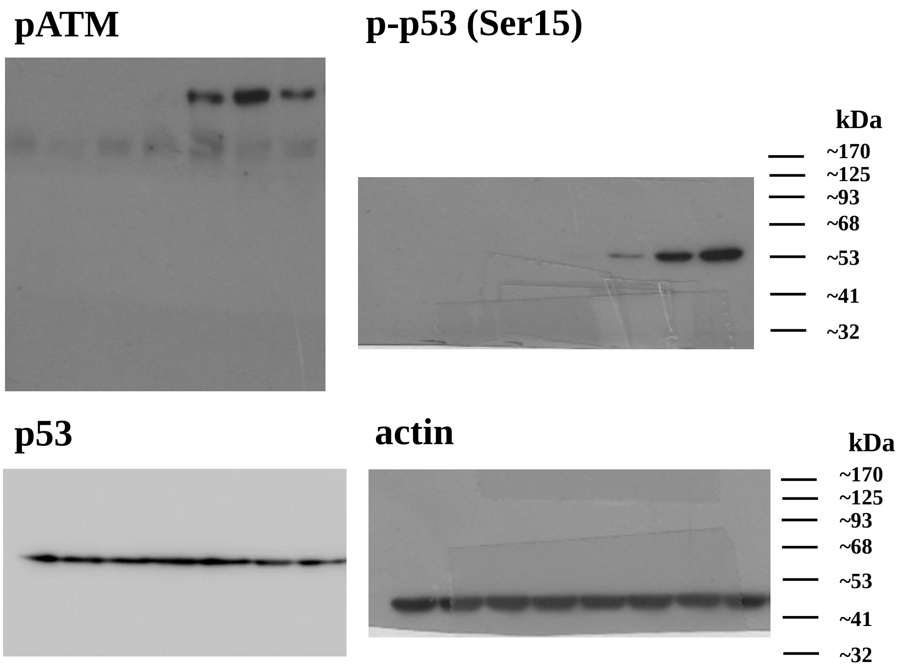

Figure 3D

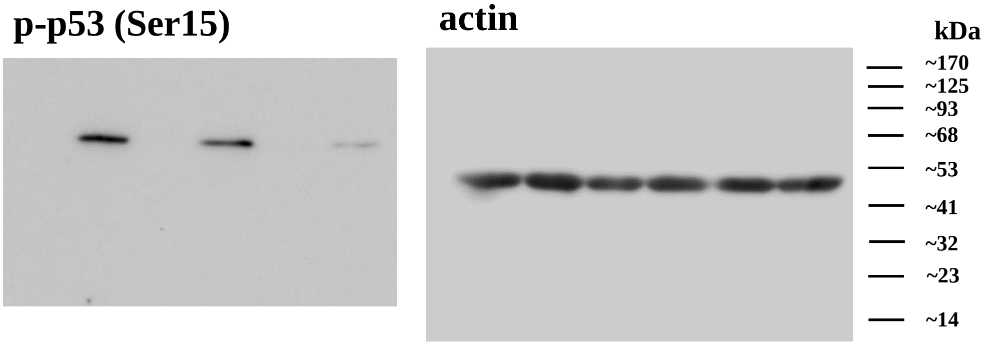

Figure 3G

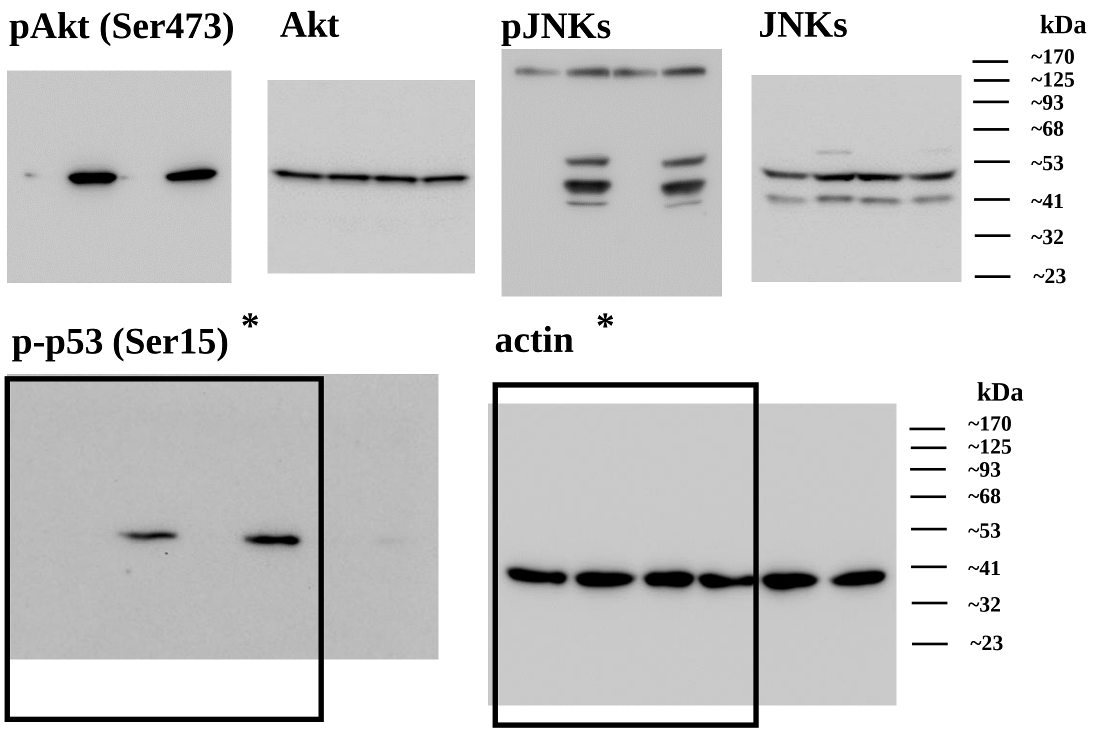

Figure 4B

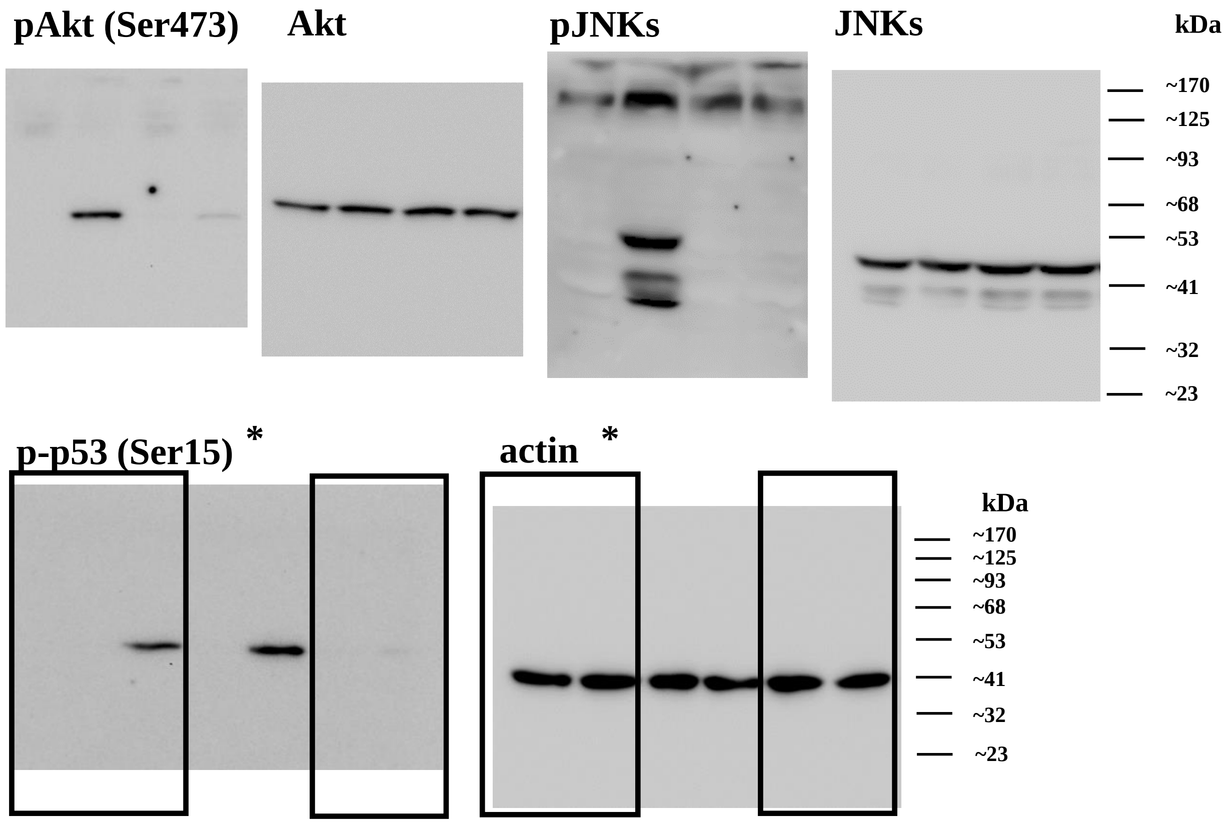

** Samples were analysed in the same SDS-PAGE and the parts of the blots marked with squares are separately presented in Figures 3G and 4B*

Figure 4E

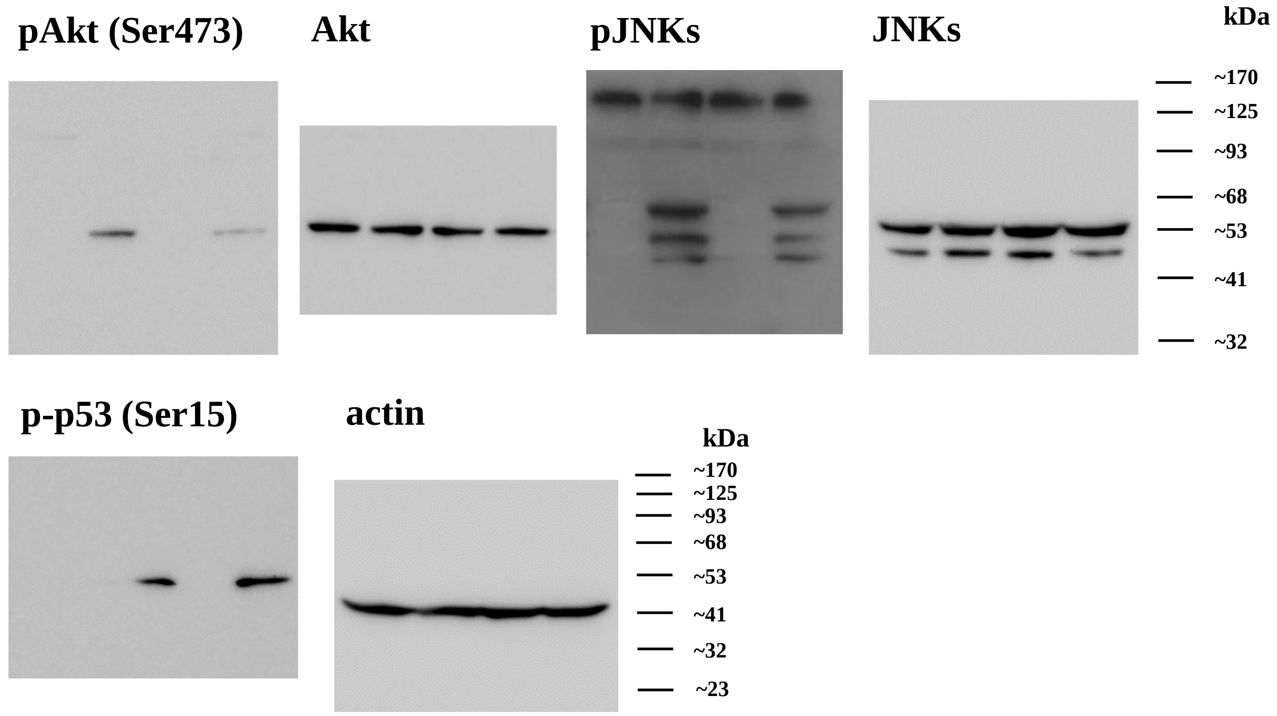

Figure 5B

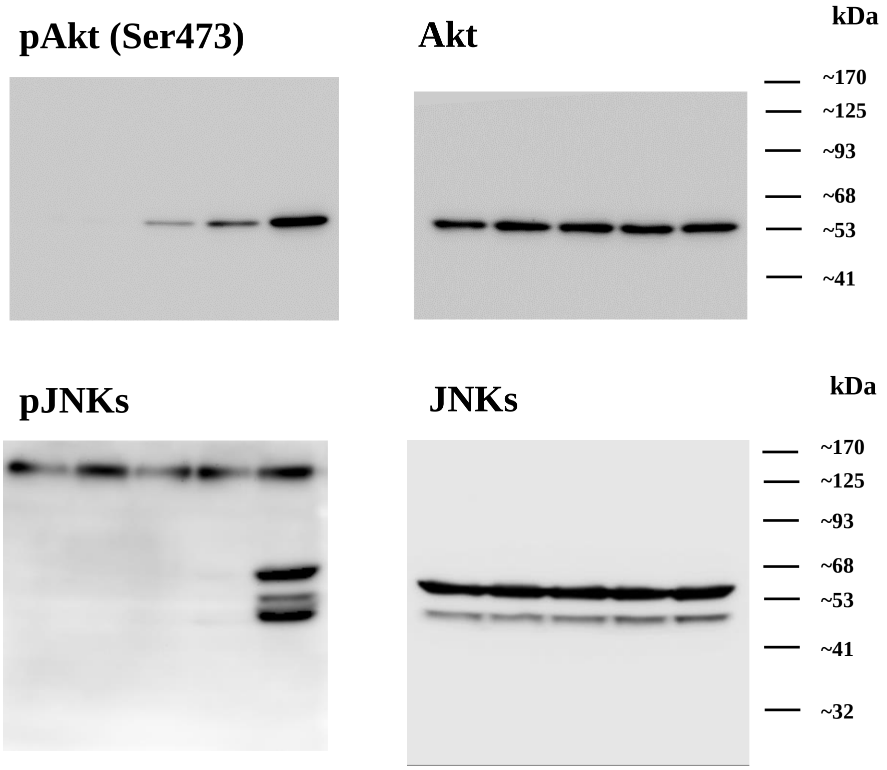

Figure 6C

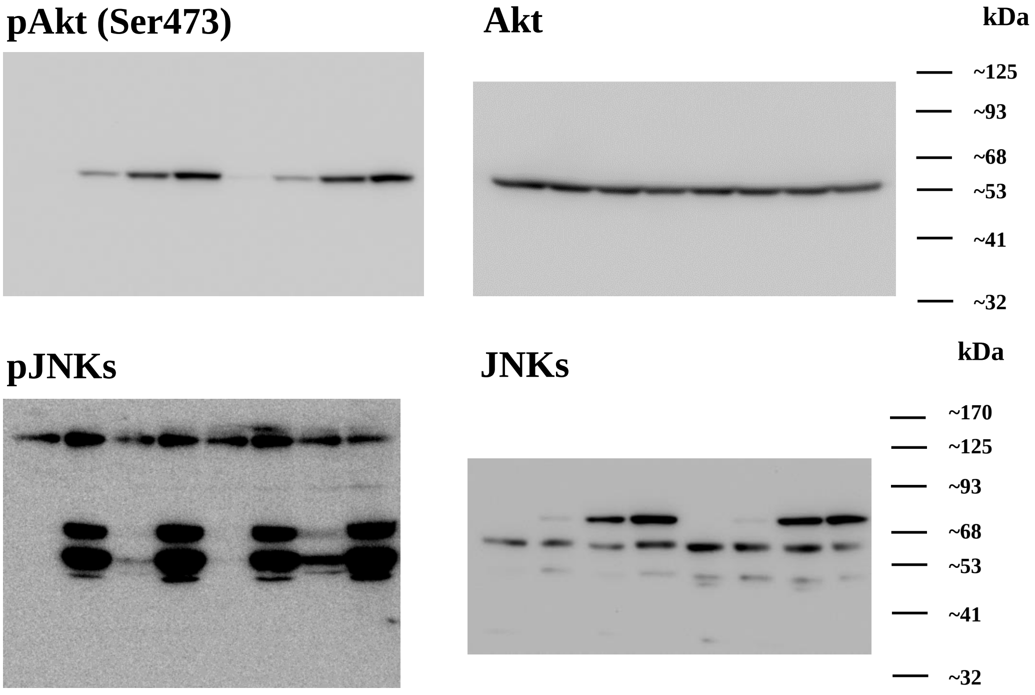

Figure 6E

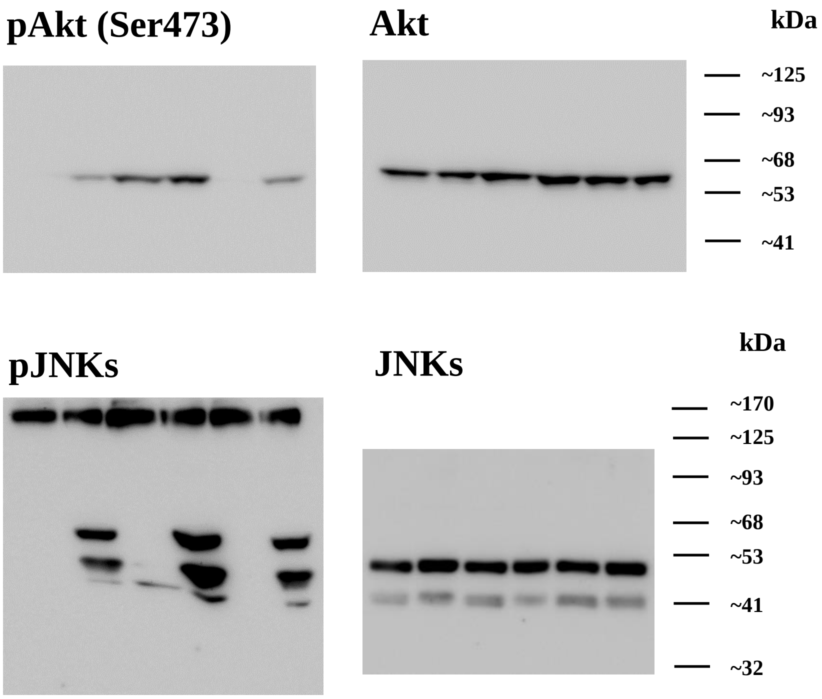

Figure 7A

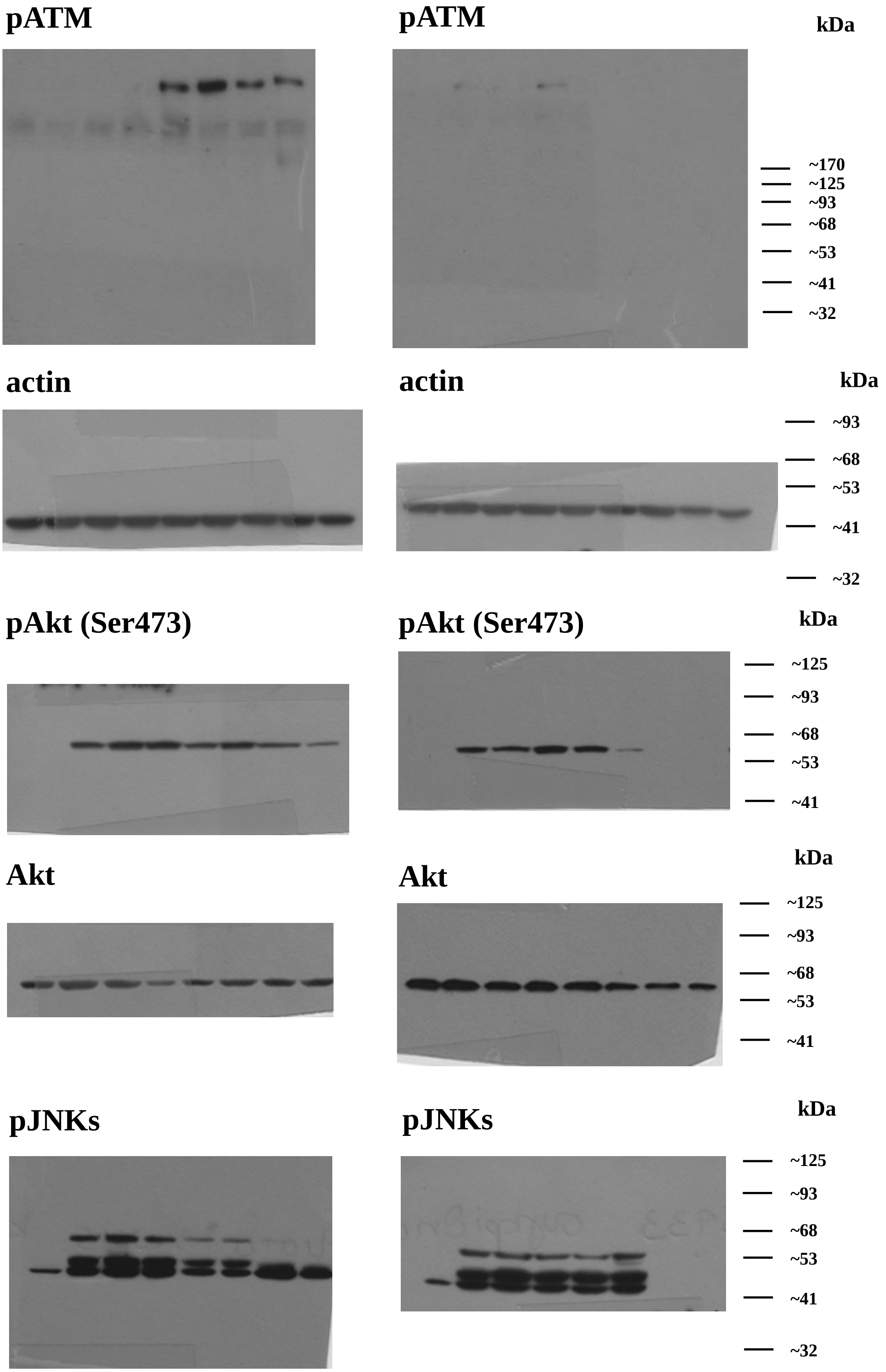
pATM
pATM
kDa
~170
~125
~93
~68
~53
~41
~32
actin
actin
kDa
~93
~68
~53
~41
~32
pAkt (Ser473)
pAkt (Ser473)
kDa
~125
~93
~68
~53
~41
Akt
Akt
kDa
~125
~93
~68
~53
~41
pJNKs
pJNKs
kDa
~125
~93
~68
~53
~41
~32

Figure 7A

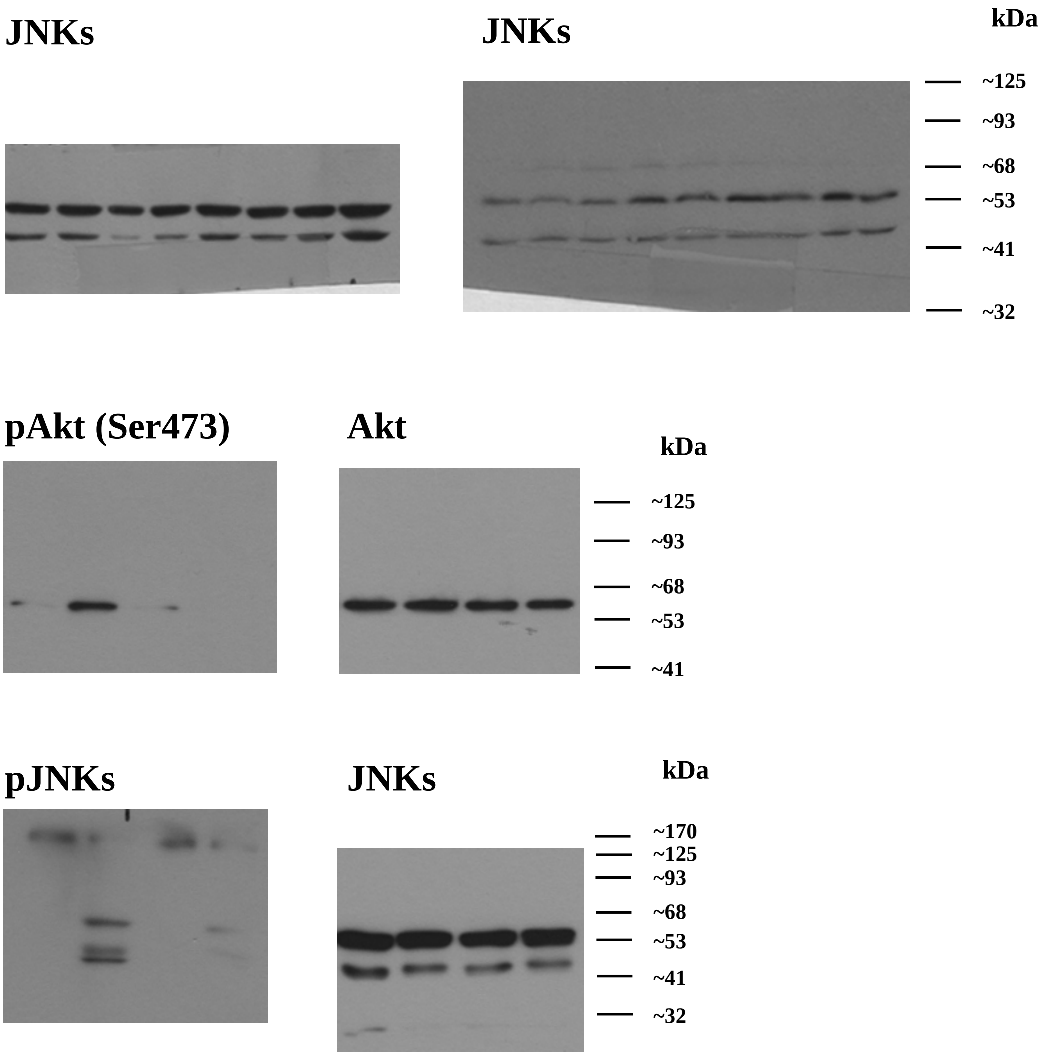
JNKs
JNKs
kDa
~125
~93
~68
~53
~41
~32
pAkt (Ser473)
Akt
kDa
~125
~93
~68
~53
~41
pJNKs
JNKs
kDa
~170
~125
~93
~68
~53
~41
~32
